# Effect of the grain arrangements on the thermal stability of polycrystalline nickel-rich lithium-based battery cathodes

**DOI:** 10.1038/s41467-022-30935-y

**Published:** 2022-06-15

**Authors:** Dong Hou, Zhengrui Xu, Zhijie Yang, Chunguang Kuai, Zhijia Du, Cheng-Jun Sun, Yang Ren, Jue Liu, Xianghui Xiao, Feng Lin

**Affiliations:** 1 Department of Chemistry, Virginia Tech, Blacksburg, VA 24061 USA; 2 Energy and Transportation Science Division, Oak Ridge National Laboratory, Oak Ridge, TN 37830 USA; 3 Advanced Photon Source, Argonne National Laboratory, Argonne, IL 60439 USA; 4 Department of Physics, City University of Hong Kong, Kowloon, Hong Kong China; 5 Neutron Scattering Division, Oak Ridge National Laboratory, Oak Ridge, TN 37830 USA; 6 National Synchrotron Light Source II, Brookhaven National Laboratory, Upton, NY 11973 USA

**Keywords:** Batteries, Batteries, Design, synthesis and processing, Energy storage, Energy

## Abstract

One of the most challenging aspects of developing high-energy lithium-based batteries is the structural and (electro)chemical stability of Ni-rich active cathode materials at thermally-abused and prolonged cell cycling conditions. Here, we report in situ physicochemical characterizations to improve the fundamental understanding of the degradation mechanism of charged polycrystalline Ni-rich cathodes at elevated temperatures (e.g., ≥ 40 °C). Using multiple microscopy, scattering, thermal, and electrochemical probes, we decouple the major contributors for the thermal instability from intertwined factors. Our research work demonstrates that the grain microstructures play an essential role in the thermal stability of polycrystalline lithium-based positive battery electrodes. We also show that the oxygen release, a crucial process during battery thermal runaway, can be regulated by engineering grain arrangements. Furthermore, the grain arrangements can also modulate the macroscopic crystallographic transformation pattern and oxygen diffusion length in layered oxide cathode materials.

## Abstract (web summary)

Enhancing the stability of positive electrodes at thermally-abused conditions is vital for next-generation lithium-based batteries. Here, the authors report in situ physicochemical characterizations to improve the fundamental understanding of the degradation mechanism in polycrystalline Ni-rich cathodes.

## Introduction

Rechargeable batteries, especially lithium-ion batteries (LIBs), play a crucial role in today’s world as energy storage devices and power sources for various applications, ranging from consumer electronics such as cellphones and laptops to electrical vehicles^1^. Despite the current success of LIBs in the market, there is a consistent pursuit of batteries with higher energy density, longer cycling life, better safety, and lower cost. Enormous efforts have been made on LIBs based on these aspects to further improve the current applications as well as to deploy new applications^2–4^. The design of these batteries can follow the dual-intercalating rocking chair configuration, and nowadays layered transition metal (TM) oxides are considered one of the most promising cathode materials^5^. Among the known layered oxides cathode candidates to date, polycrystalline ternary Li(Ni,Mn,Co)O_2_ (NMC) displays the best overall electrochemical Li-ion storage performance due to the synergistic effect of three TMs: Ni offers a relatively high capacity while Co reduces cation mixing between Li^+^ and Ni^2+^, meanwhile, Mn improves the structural stability of the host^6^. The Ni content in NMC has been progressively increased as a stepwise strategy for a Ni-rich, Co-free ultimate purpose in the community. Higher Ni content can enhance the capacity, while lower Co reduces the use of high-cost, toxic, and socially controversial cobalt minerals^7^.

Although Ni-rich NMC active materials meet the energy density requirements at the cell level with a good rate performance, the structural instability is typically accompanied by a high content of Ni^6^. This instability results in not only severe capacity fading and short cycle life, but more importantly, the poor thermal stability of charged cathodes raises safety concerns in batteries^8^. Charging the batteries to high potentials may increase practical energy density but the cathode material becomes more reactive at a deeply delithiated state. Ni^4+^ is easier to be reduced to Ni^2+^, accompanied by oxygen release^9,10^. The released oxygen can react with flammable electrolytes, accelerate severe thermal runaway, and eventually lead to catastrophic failure, toxic combustion products, and the explosion of LIBs^8–10^. The overall temperature for a rechargeable Li-ion battery in service might be in the working temperature range, but due to the reaction heterogeneity, temperature non-uniformity is common. Local temperature maxima, far away from operating temperatures suggested by the manufacturer (e.g., 0–35 °C for phones), are likely to be reached in some regions of the positive electrode^11^. During cycling, degradation happens with accelerated self-heating rate and heat accumulation, and when approaching the end of life, batteries can reach thermal runaway conditions and maximum destructive temperature in a short period^12^. Stable operation under non-ambient conditions is needed in specific applications such as oil drilling, aerospace, and the automotive industry. For example, electronics and sensors powered by batteries could be operated in the temperature range from 60 to 120 °C and sometimes even up to 200 °C in these conditions^13^. Moreover, electronics and batteries might experience thermal abuse situations, such as accident fire in the surrounding area, making the thermal study even more practically relevant. The abuse conditions are normally defined as operation over around 150 °C for Li-ion battery cathodes in the community (e.g., over 130 °C for lithium cobalt oxide cathodes, 250 °C for lithium manganese oxide cathodes)^8^. Therefore, the thermal instability of Ni-rich NMC-based electrodes becomes an important issue that needs to be addressed to avoid the safety hazard during battery use.

Various approaches have been made to improve the stability of polycrystalline cathode materials in LIBs, such as compositional optimization of Co/Mn/Ni^9^, surface modification^14^, concentration gradient engineering^15,16^, doping^17,18^, or a combination of the above-mentioned approaches^19^. The mechanism for the improved stability is generally attributed to a delayed phase transition, suppressed oxygen evolution, and alleviated stress/strain and microcrack propagation^19,20^. We noticed that the microstructure engineering of primary particles (e.g., grain shape and arrangement inside secondary particles) are understated or overlooked in the literature. The microstructure engineering of single grains inside polycrystalline materials was reported majorly as an outcome of concentration gradient^15^ or doping^18^, instead of an independent controlling factor during cathode design and synthesis. Moreover, the contribution of improved stability in these materials was usually discussed as a combined effect of microstructural and compositional engineering without detailed decoupling of these aspects. Understanding the major contributing factors for the thermal instability and fundamental degradation mechanism is essential for more accurately tailoring material properties. Oxygen release, as an important process causing the thermal instability, can be potentially regulated through engineering grain arrangements since the microstructure can govern the stress distribution and gas release pattern in NMC cathodes^21,22^. Therefore, grain microstructure has a forgotten yet important role in the thermal stability of polycrystalline cathode materials.

In this study, we investigate how grain arrangements inside secondary particles can determine the thermal stability of NMC active materials using two Ni-rich NMC cathodes with different grain shapes and arrangements as the materials platform. 3D visualizations, based on in situ synchrotron X-ray nano-tomography, show that grain arrangement governs the particle morphological evolution under thermal abuse conditions. Combining 3D nano-tomography with X-ray Absorption Near-Edge Structure (XANES) spectroscopic imaging, we reveal the Ni redox distribution and propagation in a temperature range of 25 to 250 °C, which directly reflects the 3D oxygen release patterns in NMC particles with different grains. Moreover, in situ diffraction during heating tracks the distinct macroscopic crystallographic transformation of NMC powders with different grain arrangements. Based on these measurements and analyses, we propose that increasing the oxygen diffusion length by disturbing the grain orientation and increasing the grain boundary density can be an effective solution to alleviate the thermal issues in Ni-rich Li layered oxides. We then validate this hypothesis by electrochemical measurements on cells with different types of NMC cathodes. In summary, our systematic in situ multiprobe, multiscale study highlights that tailoring grain arrangement can modulate the thermal stability of polycrystalline cathode materials in rechargeable batteries.

## Results

### Physicochemical characterization of NMC-based electrodes with different grain arrangements

**Fig. 1:**
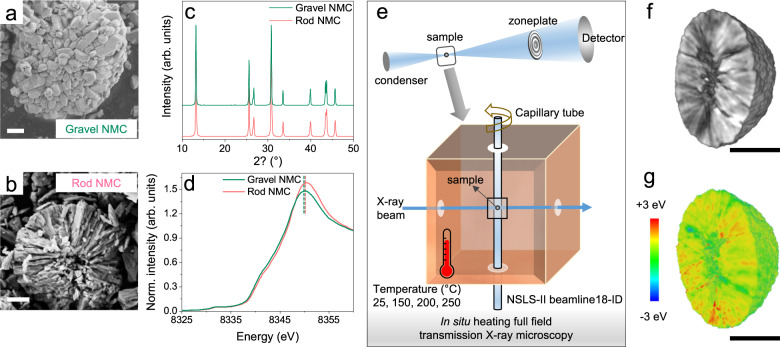
NMC with different grain arrangements and in situ X-ray nano-tomography experiment on secondary particles. SEM images of (**a**) gravel- and (**b**) rod-NMC secondary particles showing the interior grain arrangements, with a scale bar of 1 μm. **c** SXRD patterns and (**d**) K-edge XANES of gravel- and rod-NMCs in the pristine state. Vertical dash lines indicate Ni white-line energy position. **e** Schematic of the experimental setup for in situ XANES-3DTXM measurements, and the representative 3D rendering of (**f**) interior morphology. **g** Ni white-line energy distribution from XANES-3DTXM of a secondary particle, with a scale bar of 5 μm. The Ni white-line energies are color-coded, as blue and red stand for low and high oxidation states, respectively.

The scanning electron microscopy (SEM) images of the two Ni-rich layered oxides used in this study are shown in Fig. 1a, b, more morphological details can be found in Supplementary Figures 1 and 2. Both pristine cathode powders have spherical secondary particles of similar size, while the interiors of these secondary particles show distinct grain arrangements. The one with radially packed rod-shaped grains is referred to as rod-NMC, while the one with randomly packed gravel-shaped grains is referred to as gravel-NMC thereafter. The grain and atomic alignments of rod-NMC are also demonstrated in previous research work by other electron microscopy probes^16,19,21^, while Li-ion channels (diffusion pathways) along the longitudinal direction of the rod grains are confirmed by scanning transmission electron microscopy high-angle annular dark-field (STEM-HAADF)^21^.

Except for the different grain shapes and arrangements, gravel- and rod-NMC active materials share similar characteristics in crystallography and chemistry. Synchrotron X-ray diffraction (SXRD) in Fig. 1c proves both pristine NMC powders have $R\bar{3}m$ type *hkl* reflections, and simultaneous Rietveld refinement on both SXRD and neutron diffraction (ND) patterns confirms the crystallographic similarity between these two NMC active materials. A representative refinement is shown in Supplementary Figure 3, with detailed crystallographic information in Supplementary Table 1. The atom occupancies after refinement agree with inductively coupled plasma-mass spectrometry (ICP-MS) measurements (Supplementary Table 2), showing our Ni-rich NMC active materials have a Ni content of ~80%, while rod-NMC has slightly higher Co but lower Mn content compared to the gravel counterpart. The K-edge XANES of Co and Mn in Supplementary Figure 4, and Ni in Fig. 1d prove the overall oxidation states for Ni, Co, and Mn are the same for both pristine NMC materials.

Supplementary Fig. 5a shows the first charge voltage profile of non-aqueous Li metal coin cells assembled from these two NMC-based electrodes have a nearly identical curve and comparable capacities after the first charge of 2.5–4.5 V, at 25 °C and 40 mA/g (0.2 C). The comparison of Ni, Co, and Mn K-edge spectra between the pristine and charged state in Supplementary Fig. 6 shows Ni has a clear edge energy shift while Co and Mn have a shape change but no clear edge energy variation, suggesting Ni is the major redox-active element in our NMC-based electrodes at 2.5–4.5 V. Therefore, our following XANES-based experiments focused on Ni. To further compare the gravel- and rod-NMC active materials after the first charge, full-field transmission X-ray microscopy (TXM) was utilized, and the setup is illustrated in Fig. 1e. The X-rays used in our TXM measurements have energies around Ni K-edge (~8333 eV) and thus can penetrate through few-layer particles, enabling non-destructive 3D visualization of morphology within the sample. Figure 1f shows the 3D Ni absorption mapping of a charged NMC secondary particle measured at the post K-edge energy region, the absorption contrast is a reliable probe for visualizing interior morphology non-destructively. Hereafter, we refer three-dimensional TXM measurement as 3DTXM, which has a nominal spatial resolution of 30 nm in this work. Moreover, 3DTXM can be conducted in XANES mode (XANES-3DTXM), offering information on TM oxidation states. In this work, the fitted white-line energy from Ni XANES spectra was used as an indicator of the local state of charge (SoC) in the cathodes, validated by previous research work^23^. An example of the Ni oxidation state mapping deriving from XANES-3DTXM datasets can be found in Fig. 1g. To monitor the real-time morphological and chemical evolution in thermally abused conditions, we further performed the XANES-3DTXM when heating the charged NMC powders from 25 °C to 250 °C, giving us five dimensions (3D spatial + energy + temperature/time) to characterize our materials. The schematic of in situ heating XANES-3DTXM was shown in Fig. 1e, while the in situ datasets will be discussed in detail in the following sections. In this study, we measured three particles at different locations in the heating chamber independently for each type of NMC material, all particles showed similar behaviors as a function of temperature. A more detailed description of this technique, experimental setups, and explanation of temperature range selection for the in situ measurements can be found in Supplementary Note 1 and previous research works^24–27^.

### In situ chemical characterization of the NMC secondary particles

**Fig. 2:**
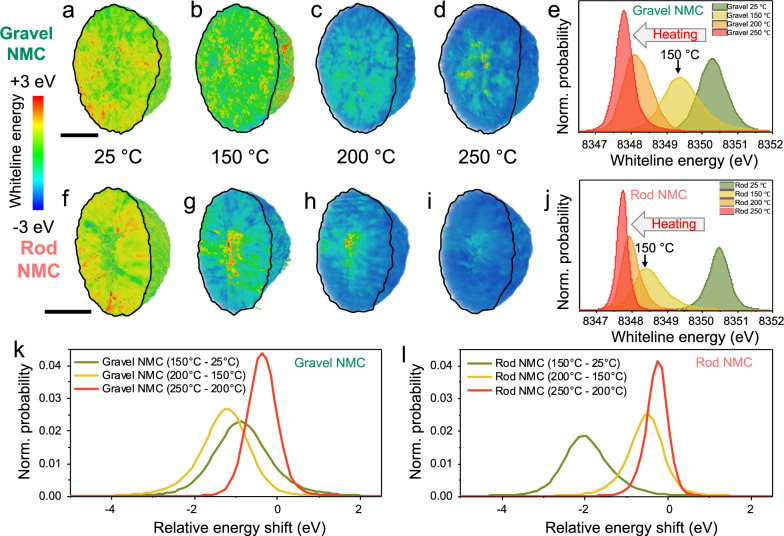
In situ XANES-3DTXM on NMC secondary particles after first charge. **a**–**d** 2D cross-section and 3D rendering of a charged rod-NMC particle showing the interior Ni white-line energy distribution at different temperatures, with a scale bar of 5 μm. **e** The SoC histogram of white-line energies at each temperature. Charing performed on coin cells between 2.5 and 4.5 V, at 25 °C and 40 mA/g (0.2 C), using lithium metal as negative electrode. The same set of plots for rod-NMC are shown in **f**–**j**. The Ni white-line energies are color-coded, where blue and red stand for low and high oxidation state, respectively. Histograms of SoC difference maps for (**k**) gravel-NMC and (**l**) rod-NMC.

Ni chemistry and oxidation state inside the secondary particles can be unveiled and analyzed as a function of temperatures based on our designed experiment. The 2D cross-section and 3D visualization of Ni oxidation state distribution inside a gravel-NMC particle can be found in Fig. 2a–d, while the histogram for each 3D dataset is shown in Fig. 2e. The same set of plots for rod-NMC particle are shown in Fig. 2f–j. The overall SoC is similar in both NMC materials at 25 °C, evidenced by bulk level Ni K-edge XANES (Supplementary Fig. 5b), as well as the SoC histograms at single particle level (Supplementary Figure 5c). Moreover, we observed a large degree of SoC heterogeneity in both particles in the charged state and noticed the 3D distribution of SoC nanodomains is different between these two NMC active materials at 25 °C (Fig. 2a, e), which agrees with previous research work^21^.

The redox reaction propagation can therefore be investigated by quantifying the evolution of Ni oxidation states, in other words, the SoC mapping. The in situ SoC mapping in Fig. 2a–d and f–i shows that the overall white-line energy gradually decreases upon heating. The histogram for each 3D dataset is shown in Fig. 2e, j for gravel- and rod-NMC particles respectively. The mean peak position indicates the average SoC in the secondary particles, lower white-line energy suggests a less oxidized Ni state, while the peak shape/width describes the heterogeneity of SoC nanodomains. With increasing temperatures, the peak becomes sharper, suggesting the SoC nanodomains were more homogeneously distributed, as both NMC particles might reach the final equilibrium at 250 °C. The overall SoC difference between these two particles is negligible when heated to 250 °C, with the same white-line mean energy of 8347.8 eV. The Ni reduction upon heating attributes to the unstable layered structure at charged state, and this structure prefers to form a more stable spinel phase, lowering the Ni oxidation state^9,21,22^. This Ni reduction and the following phase transition revealed by XANES-3DTXM at mesoscale secondary particle level is accompanied by oxygen release in Ni-rich cathodes.

Although the gravel- and rod-NMC particles show similar SoC at 25 °C and a maximum 250 °C, the SoC evolutions from 25 °C to 250 °C are quite different from each other. When heating to 150 °C, the rod-NMC histogram shows a larger energy drop of 1.9 eV, while for gravel-NMC the shift is only 0.9 eV. When heating from 150 °C to 200 °C, the rod-NMC shows an energy drop of 0.4 eV, while for gravel-NMC the shift is 1.3 eV. We used an integration method to estimate the Ni oxidation state as a function of white-line energy, based on Ni K-edge spectra measured at different charged states from previous research works^28,29^. The corresponding oxidation state for the mean energy value of each histogram in Fig. 2e, j is shown in Supplementary Table 3, and 3D renderings of the estimated Ni valence state distribution in gravel- and rod-NMC at 150 ˚C are shown in Supplementary Fig. 7, which agrees with previous research works^23,30^. Such oxidation state evolution dissimilarity, resulting from different grain arrangements, can be better appreciated by statistical analysis of the SoC difference mapping as shown in Fig. 2k, l. For example, each voxel of gravel-NMC SoC volume at 150 °C is subtracted by the voxel at 25 °C, and the histogram of the SoC difference map is shown as the green curve in Fig. 2k. The histograms suggest the gravel-NMC particle experienced moderate Ni reductions from 25 °C to 150 °C (0.9 eV shift) and from 150 °C to 200 °C (1.3 eV). In contrast, the rod-NMC particle experienced a greater Ni reduction when heated to 150 °C (1.9 eV), while a mild reaction from 150 °C to 200 °C (0.4 eV). From 200 °C to 250 °C, two types of particles had similar Ni reductions, suggesting a similar reaction mechanism above 200 °C. These observations suggest inside the secondary particle, gravel-NMC active material presented a continuous transition and Ni reduction up to 200 °C, while for rod-NMC the Ni reduction and oxygen release were faster in the relatively low temperature range, and it happened/finished with a lower thermal input, giving strong evidence that rod-NMC active material is chemically unstable compared to gravel-NMC under such thermal conditions.

### In situ structural characterization of the NMC active materials during heating

The XANES-3DTXM focused on the local and mesoscale study of NMC particles, and a macroscale crystallographic study is required to better characterize these materials at another complementary length scale. Here we chose in situ high resolution ND approach to reveal the NMC degradation during heating. Since neutron scattering strength is not dependent on the atomic number, the effect of light elements (Li and O in our case) can be observed in the presence of heavy ones in the ND patterns. Moreover, the diffractometer in this study can offer a wide *Q*-space coverage (~2.0–25.0 Å^−1^ in our case), giving accurate refinement results (e.g., lattice parameters, phase percentage, Li/TM mixing). We collected a large quantity of chemically delithiated NMC powders to meet the need for neutron experiments and to eliminate the negative impacts of inactive electrode components (i.e., carbon black, binder) and H-containing electrolyte residues on the quality of ND data (see Supplementary Note 2 for more discussion). To validate the use of chemically delithiated NMC powders as a replacement for electrochemically charged cathodes, ICP-MS was conducted on the chemically delithiated powders (Supplementary Table 2), showing a lithium removal amount of ~70%, on the same scale as the estimated amount of lithium removal by electrochemical processes. A comparison of SXRD between chemically delithiated powder and the electrochemically charged electrode is shown in Supplementary Fig. 8. These two forms of samples share similar reflection features on the SXRD pattern, suggesting the crystal structure are alike. Based on these measurements and comparisons, we speculate that the in situ ND behaviors of chemically delithiated NMC powders are representative (albeit not identical) to electrochemical cycling at intermediate rates.

**Fig. 3:**
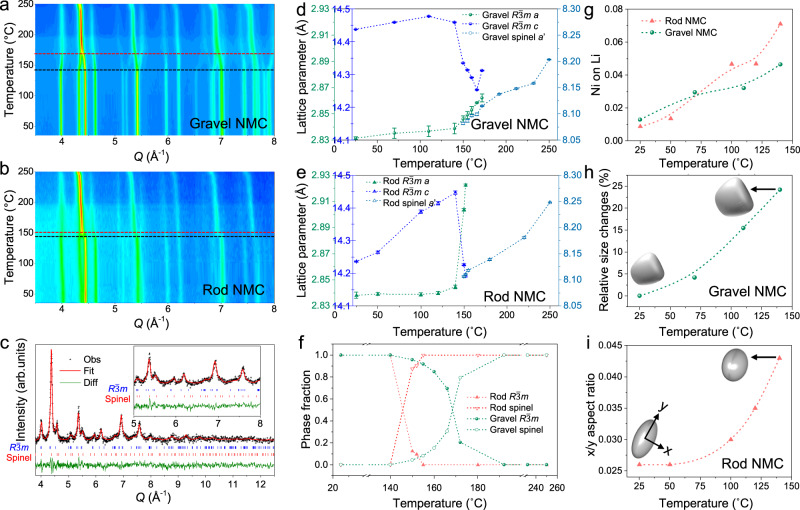
In situ neutron diffraction on delithiated NMC powders. Contour plot for in situ ND patterns of (**a**) gravel- and (**b**) rod-NMC during heating. **c** A representative refinement for gravel-NMC at 160 °C. The lattice parameters’ evolution of layered and spinel phase for (**d**) gravel-NMC and (**e**) rod-NMC, and (**f**) phase fraction changes in both NMCs. **g** Li/Ni cation mixing. **h** Relative grain size changes of layered phase for gravel-NMC, and (**i**) aspect ratio changes for rod-NMC as a function of temperature. Grain changes are enlarged on the schematics for better illustration. The error bars represent the standard deviation from Rietveld refinements. Some error bars are smaller than the symbols.

Figure 3a, b are the contour plots of in situ ND patterns for gravel- and rod-NMC powders respectively, showing distinct behaviors during heating for these two materials. For gravel-NMC, a layered to spinel phase transition starts at ~140 °C and finishes at ~190 °C. While for rod-NMC, the transition happens in a very narrow temperature window (from ~140 °C to 155 °C), which could be accompanied by a burst of oxygen release. The phase transition and crystal structures were studied in detail by conducting Rietveld refinement on the in situ ND patterns. Figure 3c is a representative refinement for gravel-NMC at 160 °C, using a two-phase model ($R\bar{3}m$ layered + ${Fd}\bar{3}m$ spinel). The representative refinements of low temperature patterns using single $R\bar{3}m$ model and high temperature patterns using the single spinel model can be found in Supplementary Fig. 9. The evolutions of lattice parameters of both phases are shown in Fig. 3d and e for gravel- and rod-NMC, while the phase fraction changes are in Fig. 3f. There was no evidence of rock salt formation from ND measurement in the temperature range studied.

Chemical delithiation resulted in a distinct unit cell at 25 °C and crystallographic evolution during heating for gravel- and rod-NMC powders. At ~70% delithiation, the *c* length of 14.44 Å for gravel-NMC as a result of increased interlayer spacing during delithiation, while a significantly lower *c* value for rod-NMC. As the temperature increased to ~140 °C, the structure remained as $R\bar{3}m$ for gravel- and rod-NMC, the expansion of *a* and *c* in gravel-NMC was majorly due to the thermal expansion, while for rod-NMC, a clear non-linear and anisotropic lattice expansion (especially *c* increase in Fig. 3e) in such temperature range might be due to a continuous reduction of TM (Ni in our case), which agrees well with our above-mentioned in situ XANES-3DTXM studies. When the temperature exceeded 140 °C, a new set of reflections showed up, which can be fitted well with a disordered Li_*x*_TM_2_O_4_ spinel phase. The $R\bar{3}m$ layered phase eventually transformed into this spinel structure at ~190 °C for gravel-NMC and ~155 °C for rod-NMC. Such complicated phase formation was accompanied by an abrupt lattice evolution in rod-NMC, as a consequence of oxygen and strain release. These phenomena, fully captured by in situ ND measurements, suggest rod-NMC active materials is less stable on a macroscopic crystallographic level under the thermal condition. Moreover, the large anisotropic lattice strain in rod-NMC $R\bar{3}m$ phase during heating to phase transition temperature is expected to manifest as severe microcracking, which contributes to the eventual mechanical failure of secondary particles. In addition, higher TM ion migration of $R\bar{3}m$ phase was found for rod-NMC under thermal conditions, as shown in Fig. 3g, suggesting Ni with less constraint and prone to a higher degree of atomic disorder^31^. The tabulated crystallographic information obtained from refinements is detailed in Supplementary Tables 4, 5, 6, and 7 for gravel- and rod-NMC powders at selected temperatures.

Note that since these two samples show different lattice parameters at 25 °C, it is reasonable to doubt whether these two NMC powders have the same level of Li content after chemical delithiation. Besides the ICP-MS results, we performed simultaneous refinement of SXRD and ND patterns for accurate Li occupancy in these delithiated powders and found the Li content is on the same level, as shown in Supplementary Table 4. Moreover, we analyzed the SXRD of both charged electrodes (Supplementary Fig. 10), which suggests lattice parameters are different even though these two cathodes are charged to the same stage and show the same capacity. We attribute the lattice difference after the same delithiation to the microstructure factors.

Due to the high *Q*-space coverage and scattering factor of ND in contrast to XRD, some subtle crystallographic information could be revealed after refinement. Here we focus on the relative NMC grain changes as heating from 25 °C to the starting point of the layered-to-spinel phase transition, by adopting the Scherrer and Stokes-Wilson equations for crystallite size calculation^32^. Note that the grain size obtained from ND refinement is for the “coherent diffracting domain”, which is the effective region with three-dimensional periodicity, and due to the presence of a variety of defects in NMC grains, the obtained size is typically smaller than the actual grain size. Nevertheless, the analysis of the relative grain shape and size as functions of temperature is reliable. For the gravel-NMC, an isotropic shape model was applied, and the relative grain size changes are shown in Fig. 3h. The gradual grain growth comes from the accumulated lattice expansion, as well as the defect agglomeration before the macroscopic phase transition around 140 °C. For rod-NMC, a uniaxial model, which is the simplest model for non-sphere shape, was adopted to imitate the rod grain shape. The comparison of fitting without shape constraints and with different grain shape models can be found in Supplementary Fig. 11. The uniaxial (110) shape constraint gives the best overall fit, suggesting that the rod longitudinal axis parallels to the [110] axial, along the direction of Li-ion channels. This observation is confirmed by STEM-HAADF results^21^. Figure 3i shows the aspect ratio changes of rod grains (radial over longitudinal length, $x/y$ ratio) as a function of temperature. With increasing temperature, although no phase transition happened below 140 °C, an aspect ratio increase for delithiated rod-NMC was observed, indicating a growth happened along the [001] direction (vertical to the [110] Li pathway). The [001] radial length expansion might happen at the expanse of shrinkage of [110] longitudinal length, resulting in a more rounded grain with a higher $x/y$ ratio. A possible explanation is that for rod-NMC active materials, the planes perpendicular to 003 layers are more prone to oxygen release, and easier for defect agglomeration, supported by first-principles calculations from Jung *et al*. on doped NMCs^33^. Schematics in Fig. 3h, i describe the grain shape changes in charged gravel- and rod-NMC during heating to ~140 °C. Herein, in situ ND measurements reveal that the distinct defect and lattice plane behaviors in rod-NMC even before the apparent phase transition, represented by the “coherent scattering domain” changes from refinements.

In summary, the in situ ND analysis on chemically delithiated NMC powders proves the rod-NMC active material is less stable on the macroscale crystallographic level, which attributes to the easier TM ion migration, abrupt phase transition, and significant grain size and shape changes.

### In situ investigation of morphological changes of NMC secondary particle

**Fig. 4:**
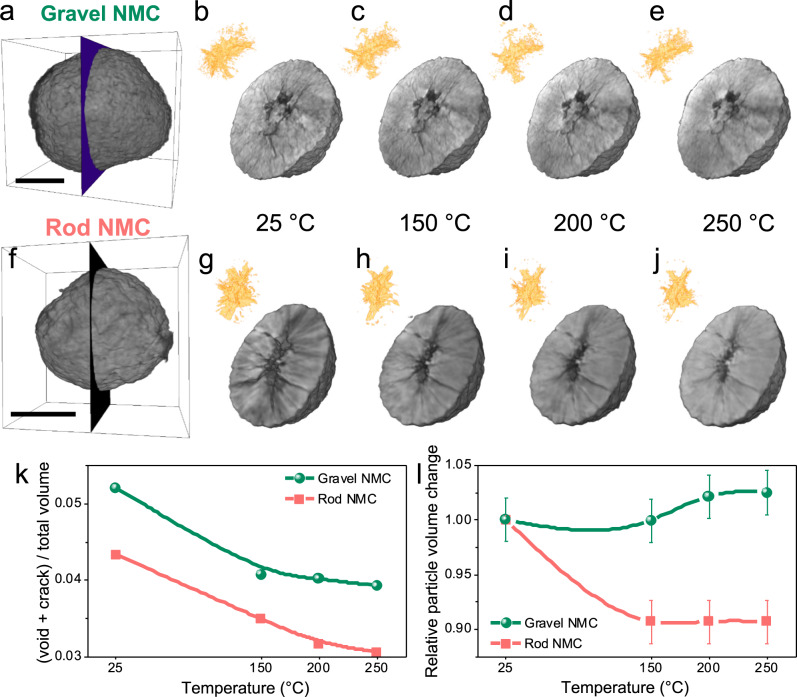
In situ synchrotron X-ray nano-tomography on charged NMC secondary particles. The 3D renderings of (**a**) a gravel-NMC secondary particle, and (**b**–**e**) the cross-section of the particle showing the interior morphology at different temperatures. The insets are 3D visualizations of partial interior voids and cracks. **f**–**j** The same set of renderings for a rod-NMC secondary particle. The scale bar is 5 μm. **k** The ratio of microcracks over the total volume of the secondary particle as a function of temperature, and **l** relative volume changes of the secondary particle with increasing temperature. Charing performed on coin cells between 2.5 and 4.5 V, at 25 °C and 40 mA/g (0.2 C), using lithium metal as negative electrode. The error bars represent the standard deviation from binarization and quantification of tomographic images.

The 3D renderings of gravel-NMC secondary particles after the first charge are shown in Fig. 4a, while the 2D cross-section of the 3D particles showing the interior morphology at different temperatures can be found in Fig. 4b–e. The same set of plots for rod-NMC particle can be found in Fig. 4f–j. Intergranular microcrack generation and propagation along grain boundaries upon the first charge at 25 °C are already observed in both NMC particles (Fig. 4b, g), which agrees with studies on different Ni-rich layered cathode materials using ex situ 3DTXM and/or cross-sectional SEM^6,20,34^. The formation and propagation of microcracks released the internal mechanical stress, which was built up during charging and discharging due to the accumulation of anisotropic structural, chemical, and charge heterogeneities^35^. Besides the microcrack propagation, we also observed a hollow interior for the secondary particles in both charged particles, which is likely a result of lattice expansion and microcrack agglomeration toward the core upon the first charge.

The disappearing of microcracks and shrinking of the hollow interior was observed upon heating in both particles. The disappearance of the voids and cracks is validated by further quantification in Fig. 4k, which shows the ratio of voids + cracks over the total volume of secondary particles gradually decreasing. There are two possible explanations. Firstly, during heating, the large microcracks generated after the first charge might decompose into smaller cracks and penetrate along surrounding grain boundaries. This could further release the accumulated inner stress, especially considering the grain boundaries are more active under a thermal input^22,36^. Since the microcrack breakdown and void invasion into grain boundaries are subtle and below the current spatial resolution, this feature cannot be picked up by 3DTXM, resulting in the apparent crack ratio decrease. Secondly, the layered NMC phase near the microcracks might have a lower oxygen release energy barrier, serving as the layered to spinel phase transition hot spot (evidenced by our above-mentioned SoC mapping). This transition led to a more porous and loosely packed low density region surrounding microcracks^37,38^, filling the gaps and decreasing the apparent crack ratio.

Although the interior structure and morphology for gravel- and rod-NMC particles shared some similarities, distinct behaviors, e.g., the collapse of the hollow interior during heating, were observed as well (gravel-NMC in Fig. 4b–e vs. rod-NMC in Fig. 4g–j). This observation is validated in Fig. 4l showing the relative volume changes of the entire secondary particle with increasing temperatures. For gravel-NMC, the secondary particle showed minimal shrinks upon heating to 150 °C, majorly due to the movements of grain boundaries and microcrack penetration towards the surface, which partially releases the inner space. Then the secondary particle had a marginal expansion, which might be a combination of intrinsic thermal expansion and the formation of a porous spinel phase. However, for rod-NMC, there was a ~10% volume shrinkage when heated to 150 °C, as a result of the collapse of the hollow interior. The in situ morphology study reveals that the rod-NMC particle suffered from severe mechanical instability at elevated temperatures (e.g., ≥ 40 ˚C) due to the hollow interior destruction. This mechanical instability can lead to particle fracturing, undermining the continuous diffusion pathways of electrons and ions, and creating fresh areas for electrode-electrolyte side reactions^34^. All these will accelerate the degradation process and exponentially deteriorate the performance of the batteries at high temperatures. In summary, the in situ interior morphology changes by 3DTXM prove that rod-NMC active materials experiences severer evolution at the mesoscale secondary particle level.

### Thermal and electrochemical characterizations of the NMC active materials

**Fig. 5:**
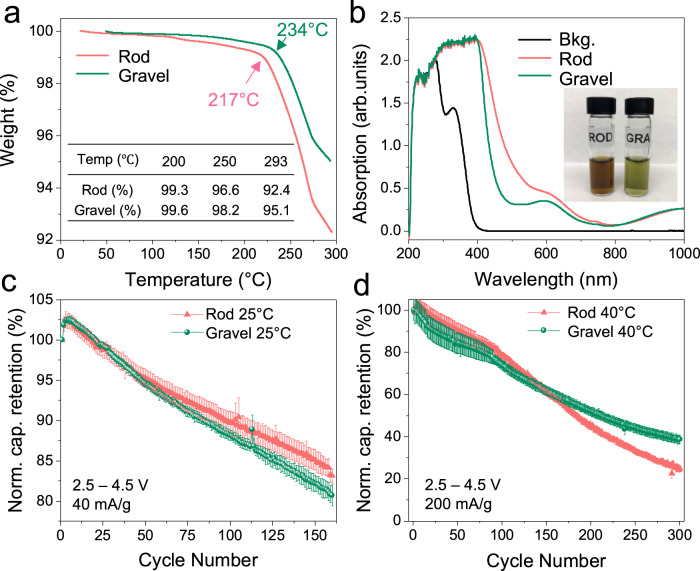
Thermal, spectroscopic, and electrochemical characterizations of the NMC materials. **a** TGA of chemically delithiated NMC. **b** UV-vis spectroscopy on chemical delithiation solution after removing the powders. **c** The normalized capacity retention of the gravel- and rod-NMCs based coin cells at 40 mA/g (0.2 C) at 25 °C, using lithium metal as negative electrode. The same set of plots for 200 mA/g (1 C) at 40 °C are shown in **d**. The error bars represent the standard deviation from three independent measurements.

The thermogravimetric analysis (TGA) of chemically delithiated NMC powders at different temperatures, shown in Fig. 5a, illustrates that gravel-NMC powder has lower overall weight loss than rod-NMC in the measured temperature range. The onset of rapid weight loss is 234 °C for gravel-NMC while 217 °C for rod-NMC, which may be attributed to the starting point of the spinel to rock salt phase transition. We did not observe a discernible buildup of the rocksalt phase from in situ ND patterns due to its low concentration. We also noticed a subtle difference after 100 ˚C, which might attribute to the formation of an unstable non-stoichiometric layered phase, and/or potential spinel phase on the surface or local domains (which cannot be detected by diffraction method), with a potential oxygen release and weight loss at the bulk level. Nevertheless, we quantified the oxygen content that remained in the samples as Supplementary Fig. 12a by assuming oxygen release is the only weight loss source during TGA. The trend is consistent with the oxygen occupancy obtained from in situ ND refinements. Moreover, DSC measurements in Supplementary Fig. 12b are consistent with the TGA analysis. The ultraviolet-visible spectroscopy (UV-Vis) spectroscopy of chemically delithiated NMC solution is shown in Fig. 5b. The rod-NMC had higher absorption between 400 and 600 nm, giving a brownish color solution, indicating severe TM dissolution after chemical delithiation in rod-NMC. ICP-MS analysis of the UV-Vis solution shows the TM ratio (Ni:Co:Mn) is 8.30:1.04:1.00 for gravel-NMC, while for rod-NMCs is 15.28:2.17:1.00, suggesting that Ni dissolution can be influenced by the grain arrangement.

All the aforementioned results from thermal experiments (XANES-TXM, ND, and TGA) strongly suggest grain arrangements inside secondary particles can affect the overall thermal stability of NMC-based positive electrodes, which in turn will affect the electrochemical Li-ion storage performance at elevated temperature (e.g., ≥ 40 ˚C). To validate this hypothesis, electrochemical measurements were performed on gravel- and rod-NMC based coin cells (using lithium metal as the negative electrode and a LiPF6-based non-aqueous electrolyte solution) under various conditions, the results are reported in Fig. 5c, d, Supplementary Figure 13, and Supplementary Fig. 14. The rod-NMC based coin cells had a 211.1 mAh/g discharge capacity at the second cycle in 2.5–4.5 V, and 85.0% retention after 150 cycles at 25 °C and 40 mA/g (0.2 C). On the same condition, the gravel-NMC based coin cells had a comparable initial capacity (199.4 mAh/g for the second cycle), but the capacity retention is slightly lower after 150 cycles (82.4%). These electrochemical measurements at 25 °C confirm that rod-NMC based electrodes perform better up to 150 cycles. Then a new batch of cells was tested at a higher rate (200 mA/g, 1 C) and 40 °C. After 100 cycles, the capacity decay is more rapid for rod-NMC based electrodes while the gravel one has higher capacity retention after 300 cycles. We conducted the electrochemical measurements more than three times, and the averaged results clearly show that the gravel-NMC based electrodes exhibit more robust performance at 40 °C.

## Discussion

The correlation between microstructure engineering and thermal stability for Ni-rich polycrystalline lithium-based battery positive electrode active materials was investigated in this study. It is shown that the grain shapes and arrangements inside secondary particles play an important role in the thermal stability of Ni-rich cathodes, which can impact electrochemical Li-ion storage performance at elevated temperatures (e.g., ≥ 40 ˚C). Via in situ XANES-3DTXM measurements, the real-time morphological and chemical evolution of different NMC particles between 25 and 250 °C were studied in-depth. In situ ND measurements revealed the crystallographic structure changes of the delithiated Ni-rich layered oxides during thermal decomposition. As in situ 3DTXM measurements carried out on a limited number of particles may raise concerns about reproducibility, we suggest measuring ex situ 2DTXM images in mosaic mode to capture a large number of particles (at the cost of losing 3D depth information) to further check the sample-to-sample variation. Moreover, we combined different independent probes in this study (bulk level hard XAS, ND, SXRD, TGA, ICP-MS, together with SEM and TEM), and each part can be cross validated from other sources. The degradation of Ni-rich layered cathode under thermally abused in-service conditions or stored above temperatures suggested by the manufacturer attributes to multiple factors. These can be categorized into two types of factors: intrinsic chemical and microstructural factors. The intrinsic factors include the spontaneous Ni^4+^ reduction and layered-to-spinel phase transition, accompanied by oxygen release. The microstructural factors are related to the mechanical integrity/robustness, such as large grain volume/shape change, local stress/strain, and microcracks propagation, which eventually lead to the microstructure collapse and cell disfunction.

Although researchers aim to tackle the Ni-rich cathode thermal stability issue from various directions^6,19^, research work carried out in this work is to identify the dominating factors from various contributions in a targeted temperature range, and then alter these intrinsic chemical or microstructural factors for improving thermal stability. For example, it is reported in the literature that doping Ni-rich layered structure with elements such as W, Ta or Al improves the structural stability^6^, due to the delayed phase transition and suppressed oxygen evolution as a consequence of cation ordering and defects engineering. Other modification/coating strategies focus on the microstructural factors to enhance the mechanical integrity, as well as passively postpone the oxygen release. Some modification methods simultaneously act on both aspects, such as concentration gradience or Ta-doping + microstructure engineering^6,18,19^.

Here we share some insights on the design principle of Ni-rich layered cathode based on the findings reported in the present research work. Our study illustrates that grain engineering is another effective method for altering thermal stability in Ni-rich polycrystalline cathodes in both intrinsic and extrinsic ways. The high specific energy and energy density, good rate capability, and stable performance seem not to be compatible with each other by the very nature of rechargeable batteries. For example, in our study the rod-NMC based coin cells has better rate capability due to fast Li-ion transport but suffers from severe degradation at elevated temperatures (e.g., ≥ 40 ˚C) because of fast Ni reduction and oxygen release. On the other hand, the gravel-NMC based coin cells with randomly oriented grains can result in better chemical, mechanical, and crystallographic stability at elevated temperatures (e.g., ≥ 40 ˚C), at the expense of a poorer rate performance at 25 °C. To further enhance the stability, we propose that the grain shape/size/arrangement should be optimized for a dense packing in the secondary particles, to achieve a minimal hollow interior after delithiation; an extra surface modification or core-shell design is beneficial to maintain the mechanical integrity if the additional cost is negligible.

There is no universal design guideline to design a cathode material that excels in every aspect of performance. Multiple factors should be balanced for a specific application case, and the major contributors to the most desired performance should be identified beforehand, and compromise on other aspects. Even though the incompatibility between high specific energy and energy density, good rate capability, and stable performance seems inevitable, researchers can still tune the major synthesis factors to reach the upper limit of performance for a specific use case. The temperature range of the most desired performance should be in consideration at the very beginning cathode design and synthesis stage for the formulation of positive electrode active materials.

## Methods

### Materials and synthesis

The transition metal hydroxide precursor, TM(OH)_2_ for the rod-NMC was obtained from Shuangdeng Group Co., Ltd, with a water content < 0.36 wt% and impurity elements < 500 ppm. The nominal TM ratio for Ni:Mn:Co is 0.82:0.06:0.12. The precursor was dried in a vacuum oven (MTI Corp. model DZF-6020-Series) at 120 °C for 12 h, then mixed with LiOH (Sigma-Aldrich, ≥ 99.9%) thoroughly and calcined in a tube furnace (MTI Corp. model OTF-1200X) under oxygen flow at 2.0 L/min. 5% extra LiOH was used to compensate the possible Li loss. The sample was heated from 25 °C to 460 °C with a rate of 5 °C /min, held for 2 h. Then the sample was heated to 750 °C at the same rate, following a holding period of 6 h. Later, the furnace was naturally cooled to 25 °C under constant oxygen flow to obtain the rod-NMC powders. The gravel-NMC was provided by the Cell Analysis, Modeling and Prototyping (CAMP) Facility of Argonne National Laboratory (LiNi_0.8_Mn_0.1_Co_0.1_O_2_ batch ID A-C020A, > 99.8%). Weak acid etching was used to expose the interior morphologies of the cathode particles. 100 mg cathode particles were dispersed in 50 mL boric acid (Sigma-Aldrich, ≥ 99.5%, pH = 4.0) solution with gentle agitation. The cathode mixture was centrifuged four times in ambient conditions at 4500 rpm and 10 mins each round (Thermo Scientific, model Sorvall ST 8). The powders were dried in a vacuum oven overnight. The chemically delithiated gravel- and rod- NMC powders were prepared by mixing the pristine powder with the oxidant and stirring for 24 h. The oxidant is 0.1 mol/L NO_2_BF_4_ (Sigma-Aldrich, ≥ 95%) in acetonitrile (Fisher Chemical, ≥ 99.9%). The delithiated powder was then centrifuged and washed with pure acetonitrile four times in ambient conditions to remove any NO_2_BF_4_ residual. The collected powder was then immediately transferred to a vacuum oven and dried at 80 °C for 12 h.

### Electrochemical measurements

The composite positive electrodes slurry was prepared using 90% active material, 5% polyvinylidene fluoride (Sigma-Aldrich, ≥ 99.5%), and 5% acetylene carbon black (Fisher Chemical, ≥ 99.9%., average particle size 0.042 µm) in N-methyl-2-pyrrolidone (Sigma-Aldrich, ≥99.0%). The slurry was then uniformly cast onto carbon-coated aluminum foil current collectors (MTI Crop. >99.9%, 16 µm in thickness) using the doctor blade coating method. The electrodes were dried under vacuum at 120 °C for 12 h without calendaring. The dried electrode sheet was punched into disks with a diameter of 10 mm, a mass loading of ~4.5 mg/cm^2^, and an approximately 70 µm thickness without including the current collector. CR2032-type coin cells were assembled in an Ar-filled glovebox (MBraun, O_2_ < 0.5 ppm, H_2_O < 0.5 ppm) using the electrode as the cathode, Li metal as the anode (Zhengzhou Jinghong New Energy Technology Co., Ltd, >99.9%, 450 µm in thickness and 15.6 mm in diameter), and glass fiber as the separator (Whatman 1827-047 934-AH, 435 µm in thickness and 1.5 µm pore size). The electrolyte was 1 mol/L LiPF6 (Sigma-Aldrich, ≥99.99%) dissolved in a 3:7 weight ratio of ethylene carbonate (EC, Sigma-Aldrich, ≥ 99%) and ethyl methyl carbonate (EMC, Sigma-Aldrich, ≥99.9%) with 2 wt.% vinylene carbonate (VC, Sigma-Aldrich, ≥ 99.5%). The electrolyte volume is approximately 100 µL per cell and has water content <10 ppm and acid content < 10 ppm. The electrochemical testing of coin cells was performed on an electrochemical workstation (Wuhan Land Co.). The performance data were measured under various galvanostatic discharge/charge rates in the cutoff voltage range of 2.5–4.5 V at 25 °C or 40 °C in an environmental chamber (ESPEC model BTU-133). The normalized capacity retention and Coulombic efficiency as a function of cycle number were calculated using the LANDdt software (Wuhan Land Co.). 1 C was defined as fully charging the positive electrode in 1 h, with a specific capacity of 200 mAh/g. To prepare the electrodes for ex situ SXRD and hard XAS measurements, the coin cells were first charged to 4.5 V at 25 °C and 40 mA/g (0.2 C), using lithium metal as the negative electrode, then transferred to the glovebox immediately. Inside the glovebox, the pristine and charged coin cells were dissembled, cathode disks were collected, rinsed with dimethyl carbonate (DMC, Sigma-Aldrich, ≥ 99%), dried, and then sealed in Ar-filled aluminum storage bags for transportation. The powders for in situ TXM measurements were prepared by dissembling the 4.5 V charged cell, then collecting the powders on the cathode disk. The powders were rinsed immediately with DMC, dried, and then sealed in an Ar-filled quartz capillary (Hampton Research, model HR6-128) for transportation.

### Material characterizations

The morphologies of NMC samples were investigated using LEO (Zeiss) 1550 field-emission SEM at an accelerating voltage of 5 kV. To determine the chemical composition of pristine and chemically delithiated NMC powders, the samples were dissolved in concentrated nitric acid, then ICP-MS was performed on a SPECTRO ARCOS ICP-AES analyzer. The UV-Vis absorption spectra were collected on a Cary series UV-Vis-NIR spectrophotometer from Agilent Technologies. Acetonitrile was used as the solvent and its spectrum is denoted as background. After chemical delithiation, the cathode/NO_2_BF_4_ slurry was centrifuged and ~1 mL of the top solution was collected to get the UV-Vis spectra for rod- and gravel-NMCs, respectively.

The in situ heating study using full-field TXM was performed at National Synchrotron Light Source II (NSLS-II) beamline 18-ID, Brookhaven National Laboratory. The XANES-3DTXM measurements were then conducted when holding the chamber temperature at 25 °C, 150 °C, 200 °C, and 250 °C, respectively. A scientific package, TXM-Sandbox, was used to reconstruct and align the tomographic datasets at different X-ray exposure energies^23^. K-edge white-line energy was extracted to benchmark the relative oxidation state of Ni by fitting the spectra in MATLAB with a combination of trigonometric and 2nd order polynomial functions for the best convergence. A commercial software, Amira-Avizo, was used for visualization.

The in situ time-of-flight neutron diffraction was performed on Nanoscale Ordered Materials Diffractometer (NOMAD) BL-1B, Spallation Neutron Source (SNS) at Oak Ridge National Laboratory. About 0.2–0.3 g of chemically delithiated powders were loaded into a vanadium can. The can was then mounted in a high-temperature furnace at the beamline. Neutron diffraction data were collected continuously when ramp heating from 25 °C to 250 °C with a rate of 1 °C/min under vacuum (<10^−6^ torr). The refinement of neutron diffraction patterns was conducted using the software GSAS-II^39^. Details of refinements can be found in Supplementary Note 3. XANES measurements were performed on the electrodes in transmission mode at the beamline 20-BM-B of the advanced photon source (APS) at Argonne National Laboratory. Energy calibration of each spectrum was made by aligning the first derivative maximum of a reference Ni XANES spectra collected simultaneously from the metal foils in the reference channel. Different synchrotron sources were used to collect XRD patterns for electrochemically delithiated electrodes and chemically delithiated powders. Powder XRD was performed at beamline 11-ID-C at APS with a wavelength of 0.1173 Å (105.7 keV). The powder samples were loaded in Kapton capillaries, and patterns were recorded on a Perkin Elmer flat-panel amorphous-silicon 2D detector with a collection rate of 20 s. CeO_2_ was used for calibration. Electrode XRD was performed at beamline 11-3 of Stanford Synchrotron Radiation Lightsource (SSRL), with a wavelength of 0.9762 Å and a collection rate of 0.5 s. LaB_6_ was used for calibration. A thermogravimetric analysis study was performed on a TGA Q50 in the range of 25–300 °C at a temperature ramping rate of 5 °C/min under oxygen flow.

## Supplementary information

Supplementary Information

## Data Availability

The datasets generated during and/or analyzed during the current study are provided in the Supplementary Information. Other datasets are available from the corresponding author on reasonable request.

## Code availability

The codes that support the findings of this study are available from the corresponding author upon reasonable request.
