# Supplementary material for: Effect of the grain arrangements on the thermal stability of polycrystalline nickel-rich lithium-based battery cathodes
Source: Nat Commun. 2022 Jun 15;13:3437. doi: 10.1038/s41467-022-30935-y (PMC9200779; doi:10.1038/s41467-022-30935-y)
Supplement: Supplementary file 1 — Supplementary Information [file 41467_2022_30935_MOESM1_ESM.pdf]

Supplementary Information:

Effect of the grain arrangements on the thermal stability of polycrystalline nickel-rich lithium-based battery cathodes

Dong Hou[1,#], Zhengrui Xu[1,#], Zhijie Yang[1], Chunguang Kuai[1], Zhijia Du[2], Cheng-Jun Sun[3], Yang Ren[3,4], Jue Liu[5], Xianghui Xiao[6,*], Feng Lin[1,*]

[1] Department of Chemistry, Virginia Tech, Blacksburg, VA 24061, USA

[2] Energy and Transportation Science Division, Oak Ridge National Laboratory, Oak Ridge, TN 37830, USA

[3] Advanced Photon Source, Argonne National Laboratory, Argonne, IL 60439, USA

[4] Department of Physics, City University of Hong Kong, Kowloon, Hong Kong, China

[5] Neutron Scattering Division, Oak Ridge National Laboratory, Oak Ridge, TN 37830, USA

[6] National Synchrotron Light Source II, Brookhaven National Laboratory, Upton, NY 11973, USA

[#] These authors contributed equally

* Author to whom correspondence should be addressed: fenglin@vt.edu and xiao@bnl.gov

Supplementary Note 1: XANES-3DTXM measurements

X-ray tomography measurement can be achieved by taking TXM projections of a rotating sample at each fraction of a degree. The 2D images collected at all angles are then reconstructed into a 3D volume. The hard X-rays can penetrate through the sample, enabling non-destructive 3D analysis of structure and morphology within the sample. Compared with 2DTXM which is a projection through the entire sample, the 3D structure from X-ray tomography can detect the cracks, porosity, and tortuosity within the sample with a spatial resolution of tens of nanometers.

A more powerful technique actively developed in recent years is coupling TXM with X-ray absorption near edge structure (XANES) spectroscopic capability. This is achieved by collecting and reconstructing TXM projections at a series of X-ray energies across the absorption edge of an element. A XANES spectrum can be generated at each voxel of the reconstructed 3D volume by imposing and aligning the absorption objects at different energies, which involves sophisticated image alignment in data processing. The shape of the XANES spectra represents the local chemical fingerprints of the element, therefore the 3D information on oxidation states can be achieved by fitting the spectrum on every voxel of the object. A great advantage of XANES-3DTXM over the most widely used 2D approaches is that the information is depth-averaged in 2D measurements, chemical depth profile along the beam direction is not available, while XANES-3DTXM offers the great capability for finer chemical and morphological features inside the sample.[1]

With the high flux of synchrotron X-rays and the development of optics, detectors, and algorithms for fast data acquisition, one tomography measurement could be carried out in minutes, which makes *in situ*/*operando* experiments possible.[2] This *in situ* XANES-3DTXM approach allows us to observe the real-time morphological and chemical evolution at elevated temperatures. The large field of view (20-40 μm) and reasonably high spatial resolution (~30 nm) offer the capability to analyze NMC cathodes at different length scales, ranging from voids of tens of nanometers, to cracks of hundreds of nanometers, to secondary particles of tens of micrometers. This so-called 5D mapping approach can greatly help to understand the reaction mechanisms of cathode active materials, and the quantitative analysis methodology is broadly applicable for other energy materials.

The powders for *in situ* TXM measurements for this study were prepared by dissembling the 4.5 V charged cell, then collecting the powders on the cathode disk. The powders were rinsed

immediately with dimethyl carbonate, dried, and then sealed in an Ar-filled quartz capillary. The capillary was mounted on a holder base and then inserted into the chamber. The 3DTXM was conducted by acquiring tomographic images of the particles at 519 angle positions in 180° range. The XANES-3DTXM was done by repeating the tomographic scans of the same particles in an X-ray energy range across the white-line of Ni K-edge in 1 eV step. The heating rate was 5°C/min when changing the temperature. After reaching the target temperature, a 20 mins waiting time was given to stabilize the temperature. The total XANES-3DTXM collection time was ~30 mins for one particle at each temperature.

In order to do quantification and further analysis of the morphological characteristics, the greyscale absorption mapping is normalized and then binarized using Otsu's thresholding method, which is effective in minimizing the intraclass variance of the black and white pixels after binarization.[3]

Supplementary Note 2: Neutron Diffraction

Chemically delithiated NMC powders were used for *in situ* ND study for several reasons:

- Firstly, for a better signal, the required amount of materials is large (300~400 mg). Considering obtaining such NMC powders with high purity from charged cells is very challenging, the chemical delithiation was chosen as an alternative.
- Secondly, XANES-3DTXM results show the NMC thermal instability originates from two parts: the intrinsic Ni reduction and oxygen release, as well as the morphological factors (e.g., microcracks, voids, etc.), and these two parts cannot be decoupled by solely TXM approach. Chemical delithiation can open up the secondary particles, resulting in similar cracks ratios, porosity, and tortuosity for gravel- and rod-NMC. Therefore, the intrinsic factor for the stability in NMC can be focused on, while the effect of microcracks can be ruled out.
- Thirdly, cell variation and parasitic reactions can lead to some over- or under-estimation of the absolute amount of lithium intercalated into and deintercalated from the host material for electrochemical processes,[4,5] making a direct comparison between two delithiated NMCs difficult.
- Finally, the presence of carbon black, polymer binders, and proton-containing electrolyte residual from electrochemically cycled cathodes will impact the scattering signals, making Li site occupancy calculation less convincing.

Neutron diffraction data were collected continuously from 25°C to 250°C, with a collection time of 5 mins per dataset. In addition, a longer exposure time of 30 mins was used at 25°C, 100°C, 150°C, 200°C, and 250°C for better quality. For each *in situ* dataset, six detector banks with nominal diffraction angles of 7°, 15°, 31°, 65°, 120°, and 150° were simultaneously measured.

For each sample, data from different detector banks with different *Q*-spacing coverages were analyzed simultaneously for structure refinement at each temperature.

Supplementary Note 3: Refinements on Diffraction Patterns

The average structure before heating was first investigated by performing Pawley fits on the as collected ND data. For Pawley fit the background terms, unit cell parameters, and peak profiles were refined. The results of the Pawley fit were used as starting models for the analysis using the Rietveld method where the scale factor, zero offset, phase fractions, atomic positions, occupancies, and atomic displacement parameters were also refined. The instrument parameters were calibrated by refining a standard Si powder (SRM 640e from NIST) at corresponding temperatures.

In the following analysis of *in situ* ND patterns, the refinement of phases (phase fraction and lattice parameters) and atoms (position, occupancy, and thermal displacements) is performed in the sequential order, meaning that the values obtained from temperatureT_n will be the initial values for refining at the next temperature T_{n+1}. We applied constraints to atoms on the same site to have the same thermal displacement, for example, the Li and Ni (intermixed on Li layer) on $3a$ sites have the same displacement parameter; while Li (intermixed on TM layer), Ni, Mn, and Co on $3b$ sites will share the same value. For Li/Ni intermixing ratio, we refine Ni occupancy on Li layer ($3a$ site), and the Li occupancy on the TM layer ($3b$ site) will change accordingly. The thermal displacement parameters for Li will be fixed once they exceeded the theoretical upper limit. Otherwise, we found that the refinement process can hardly converge. Ni/Mn/Co were fixed at the nominal composition obtained from 25˚C during *in situ* refinement, since the TM ratio didn't change according to our ICP-MS measurements on 25˚C and 250˚C heated samples.

For peak broadening, besides the instrumental contribution, there are two main types of contributions: the size and the strain components. The former depends on the finite size of the coherent diffraction domains and the latter is caused by any lattice imperfection (point, line, or plane defects). Here we used the Stephens model to count for the anisotropic strain distribution caused by lattice imperfection, while the Scherrer and Stokes-Wilson equations for size calculation.

Supplementary Figures:

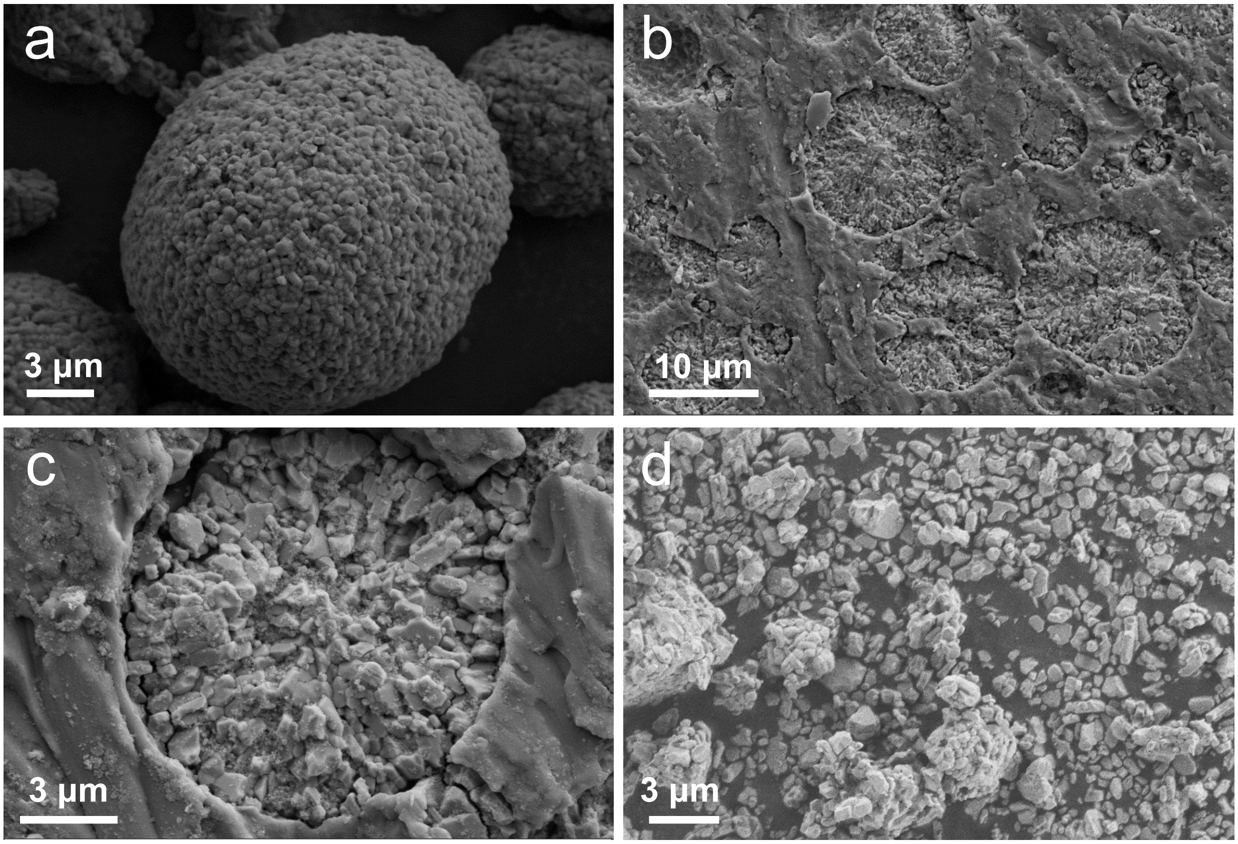

Supplementary Figure 1 SEM images of gravel-NMC. (**a**) secondary particle, (**b, c**) the interior of secondary particles after polishing, (**d**) breakdown of the grains after weak acid etching.

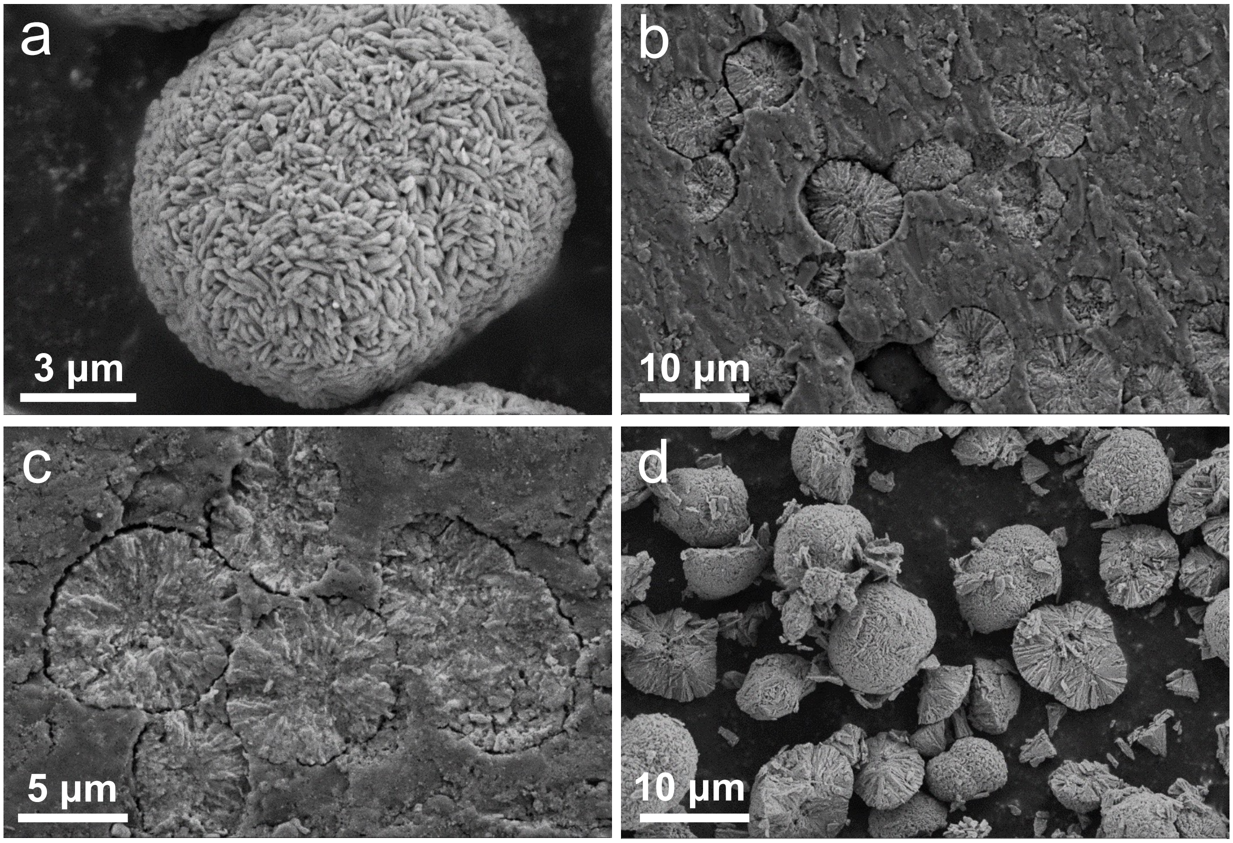

Supplementary Figure 2 SEM images of rod-NMC. (**a**) secondary particle, (**b, c**) the interior of secondary particles after polishing, (**d**) breakdown of the grains after weak acid etching.

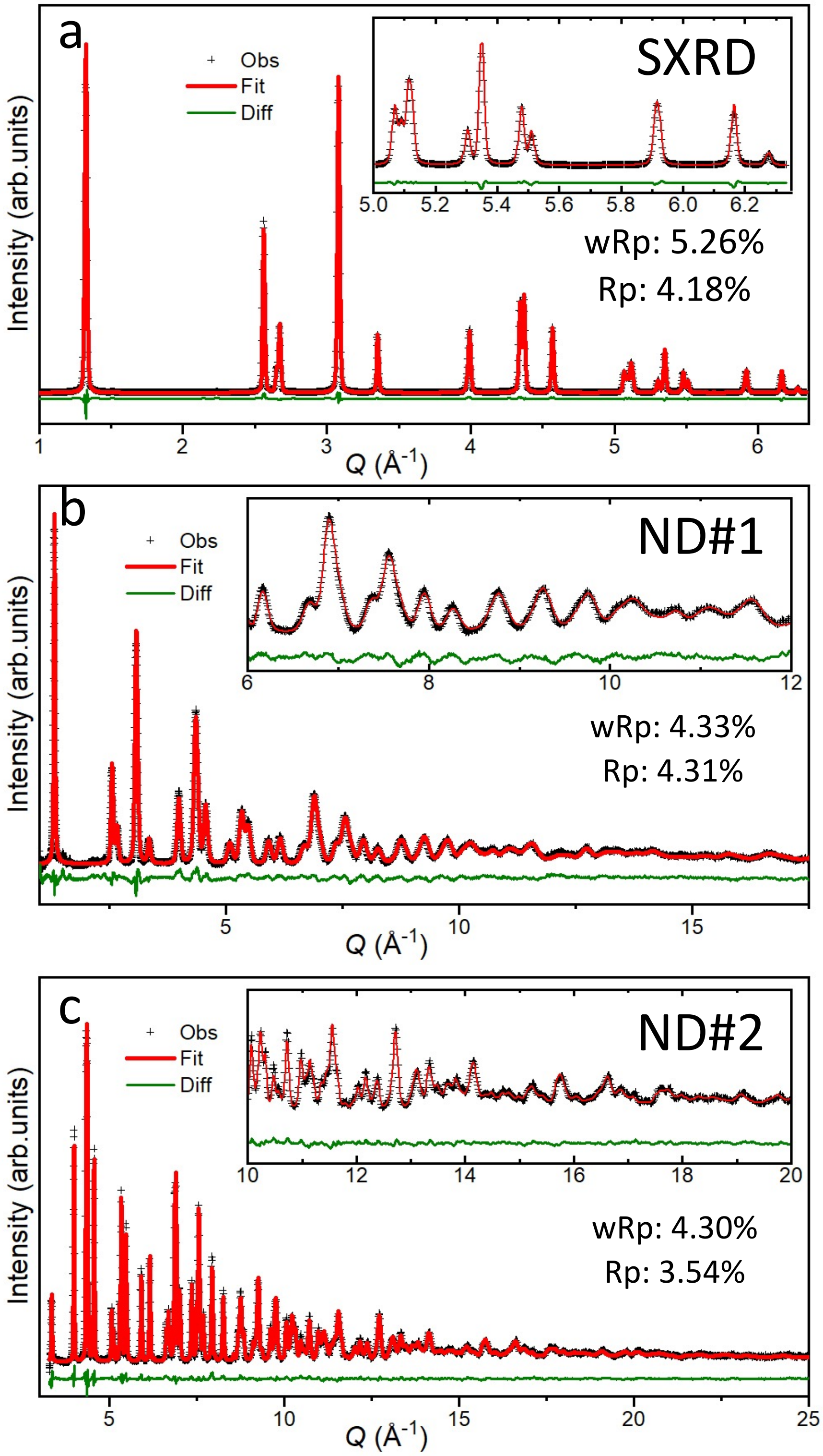

Supplementary Figure 3 Fitted patterns after simultaneous refinement using (a) SXRD and (b, c) ND datasets. Pristine gravel-NMC was selected as the example. The maximum useable *Q*-space exceeds 20Å^{-1} in panel **c**.

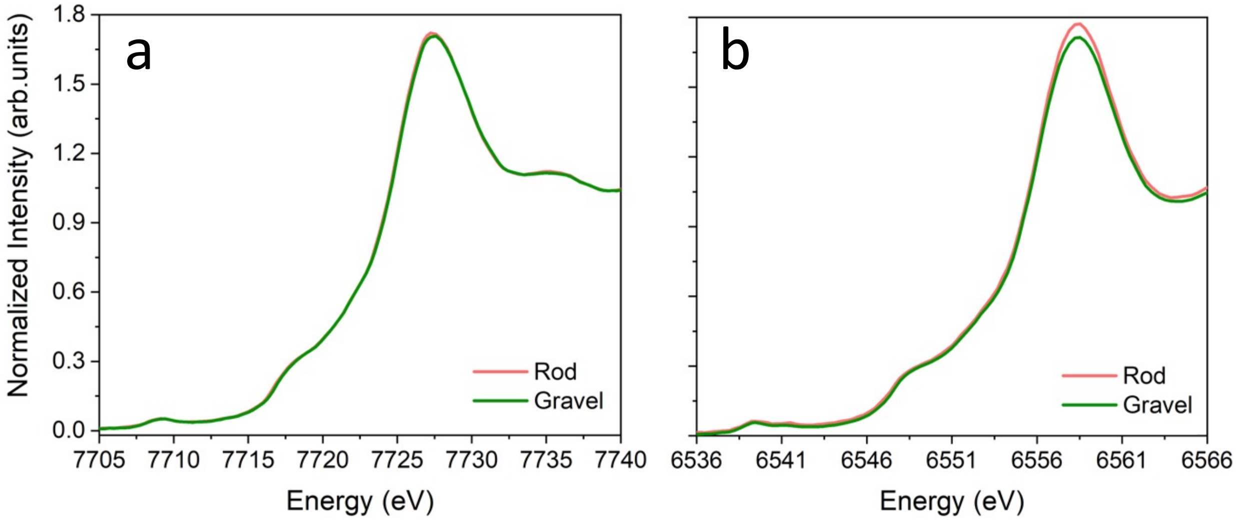

Supplementary Figure 4 K-edge XANES of (a) Co and (b) Mn for pristine rod- and gravel-NMCs. Co and Mn oxidation states are identical between these two samples.

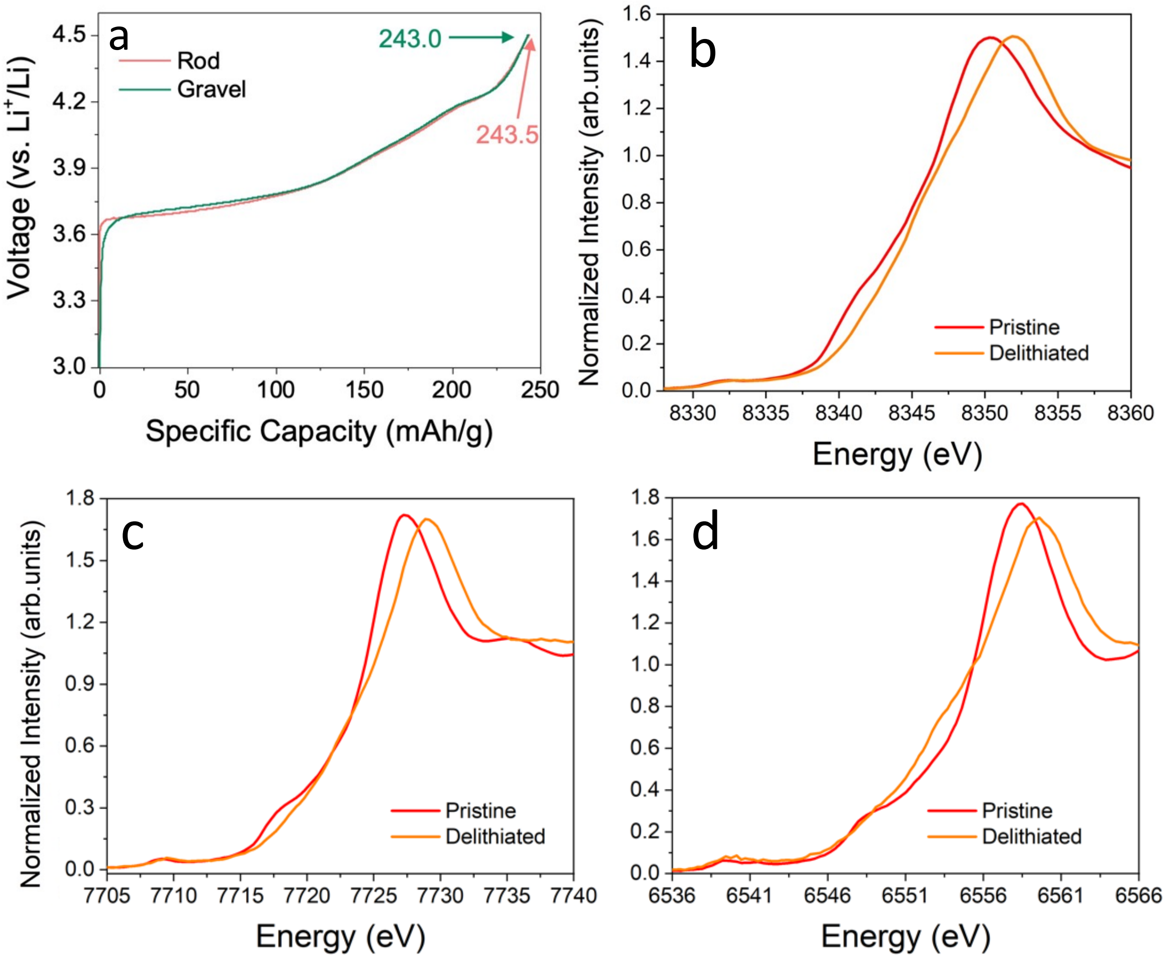

Supplementary Figure 5 (a) First charge profile of rod- and gravel-NMC based coin cells at 2.5-4.5 V at 40 mA/g (0.2C) and 25˚C after a formation cycle, using lithium metal as negative electrode. Comparison of K-edge XANES of (**b**) Ni, (**c**) Co, and (**d**) Mn between pristine and electrochemically delithiated NMCs. Ni shows a clear shift, while for Co and Mn there is a shape change but no major edge energy shift, indicating Co and Mn are not the major redox-active elements during electrochemically delithiation.

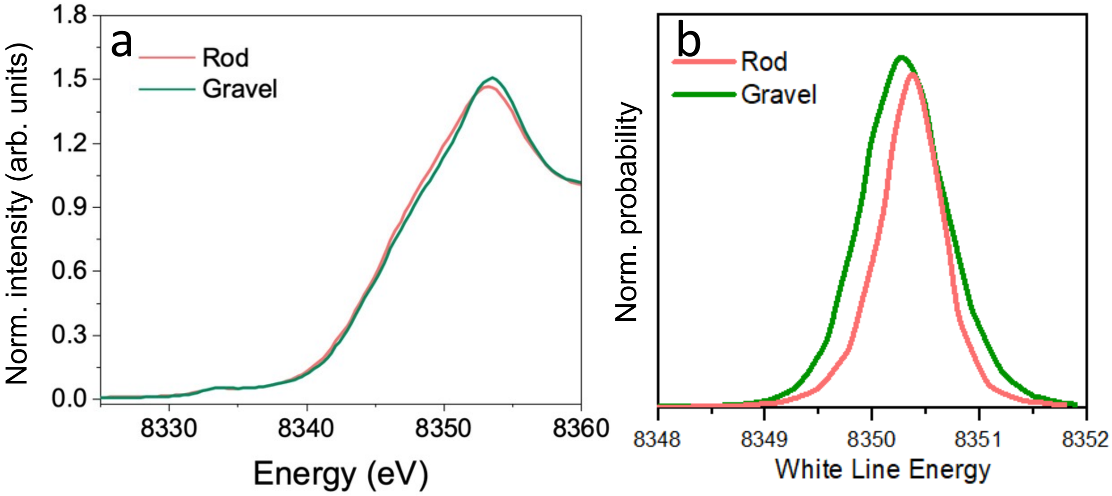

Supplementary Figure 6 (a) Bulk level Ni K-edge XANES and **(b)** statistical histogram of SoC distribution in XANES-3DTXM datasets at particle level for both NMC-based positive electrodes after first charge of rod- and gravel-NMC based coin cells at 2.5-4.5 V at 40 mA/g (0.2C), 25˚C after a formation cycle, using lithium metal as negative electrode.

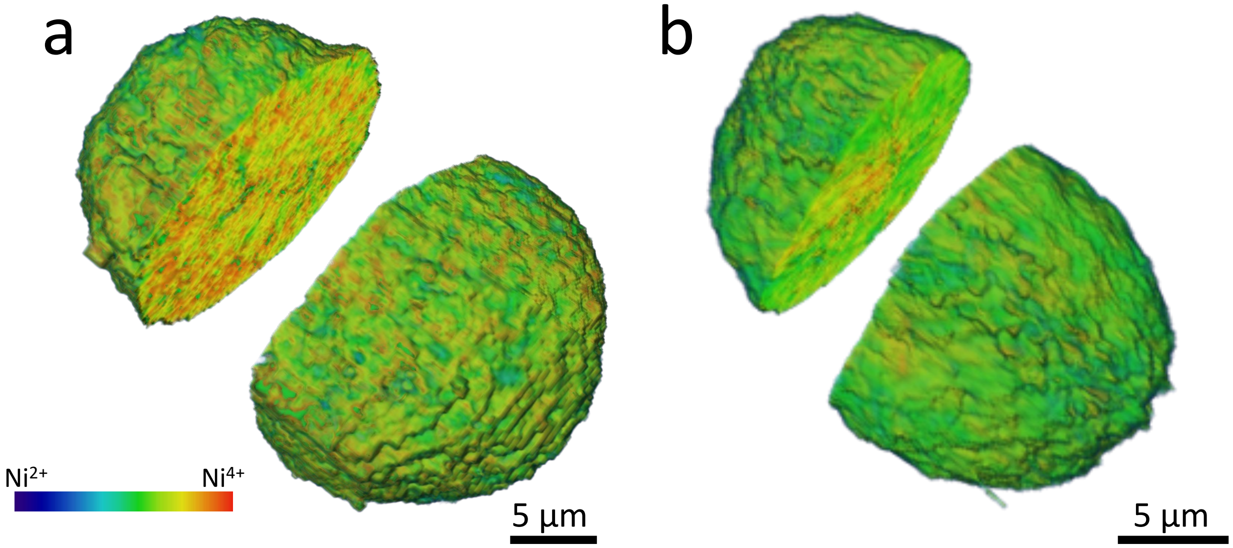

Supplementary Figure 7 Rendering of estimated Ni oxidation state distribution at 150˚C for (a) gravel- and (b) rod-NMCs. Gravel-NMCs have a higher Ni oxidation state (less reduced) at intermediate temperatures. The Ni oxidation state are color-coded, as blue and red stand for low and high oxidation state, respectively

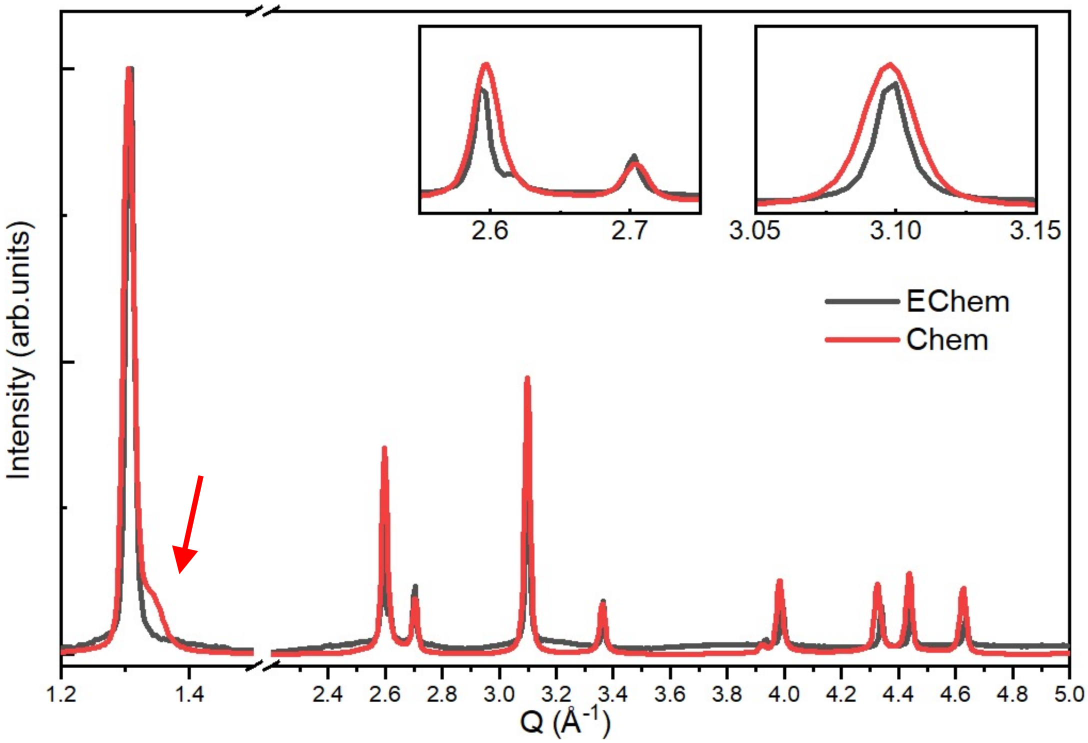

Supplementary Figure 8 Comparison of synchrotron XRD patterns between electrochemically (EChem) delithiated electrodes and chemically (Chem) delithiated powders. The red arrow indicates a H3 phase in chemically delithiated NMCs, showing chemically delithiated NMC powders is similar (but not identical) to electrochemical cycling. Gravel-NMC is used as an example, rod-NMCs follow the same manner.

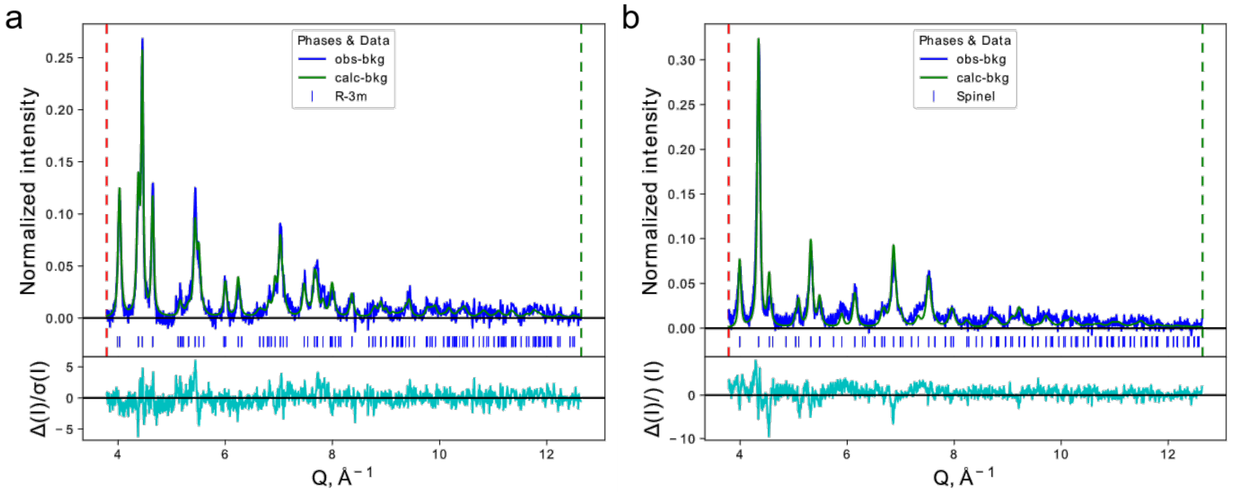

Supplementary Figure 9 Representative refinements at different temperature range. (**a**) refinement of a 50°C pattern using single $R\overline{3}m$ model. (**b**) Refinement of a 225°C pattern using single spinel model.

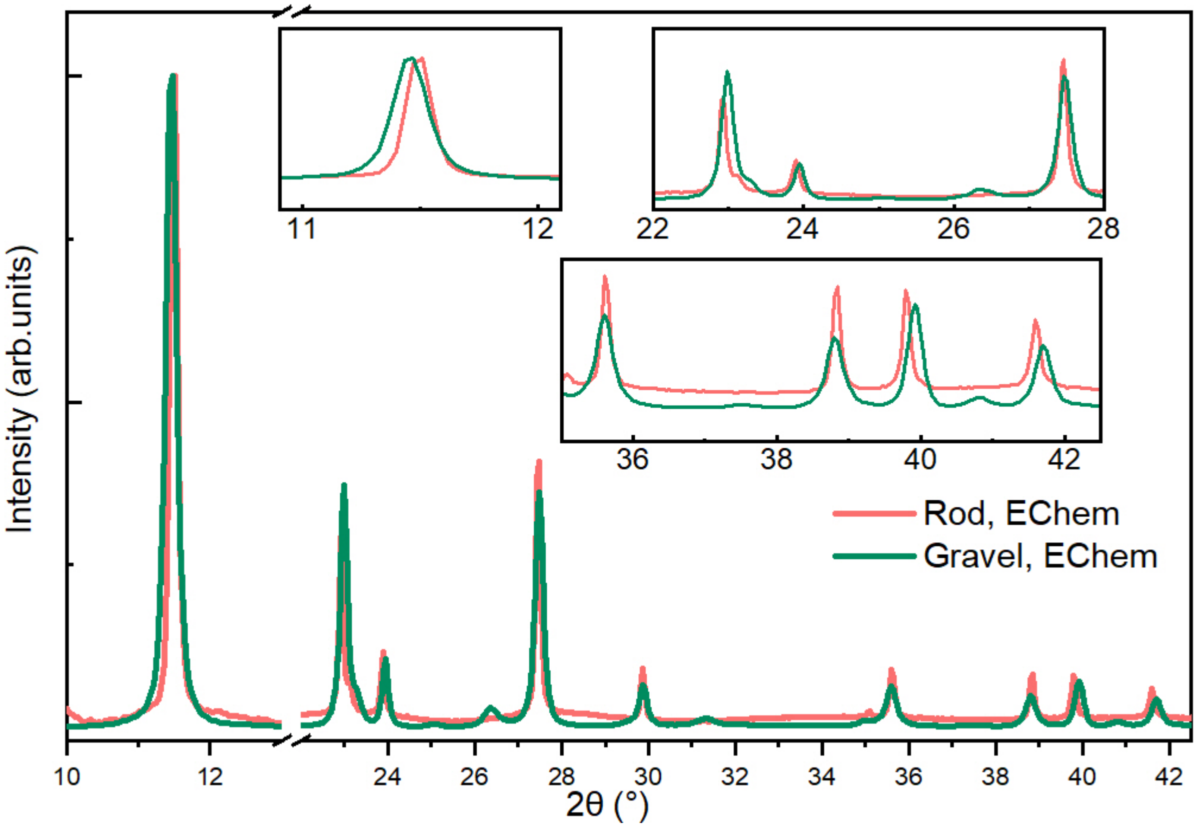

Supplementary Figure 10 Comparison of synchrotron XRD patterns between charged rod and gravel electrodes. The clear peak shift suggests a difference in lattice parameters although the delithiation is the same in two samples. Charing performed on coin cells between 2.5-4.5 V, at 25°C and 40 mA/g (0.2C), 25˚C, using lithium metal as negative electrode

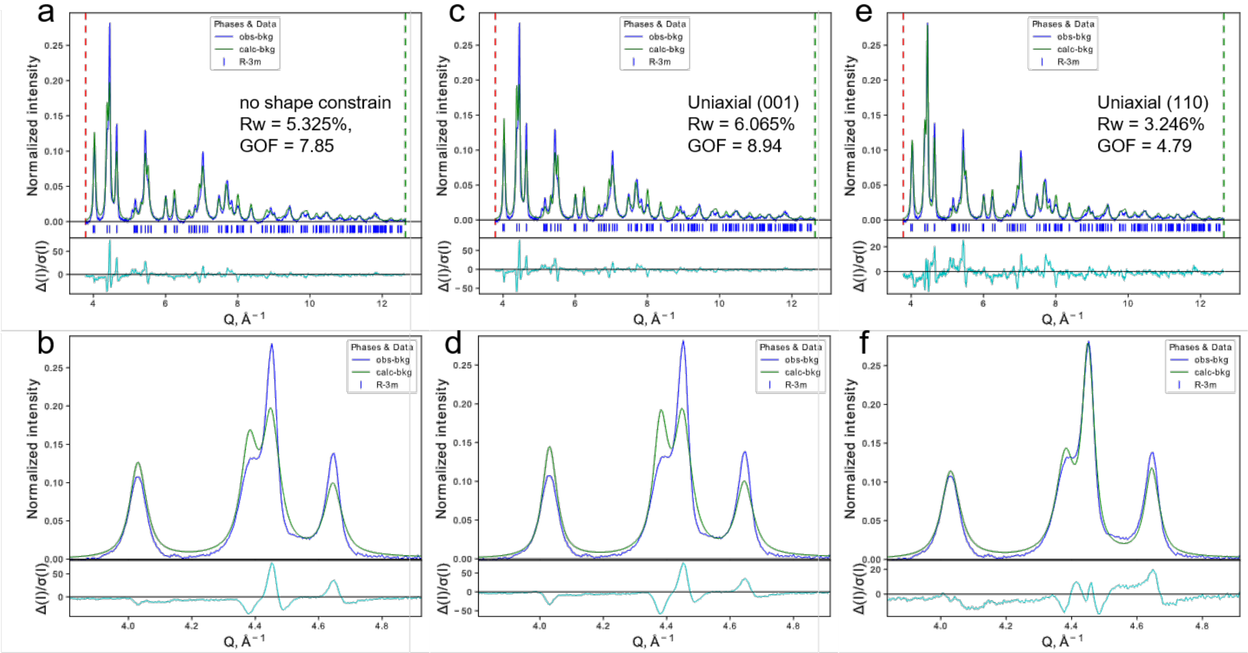

Supplementary Figure 11 Comparison of fitting using different grain shape models. (**a, b**), fit without shape constraints (**c, d**) with uniaxial (001) shape constraint, and (**e, f**) with uniaxial (110) constraint. Note the residual curves in **e** and **f** are plotted in a smaller scale than others.

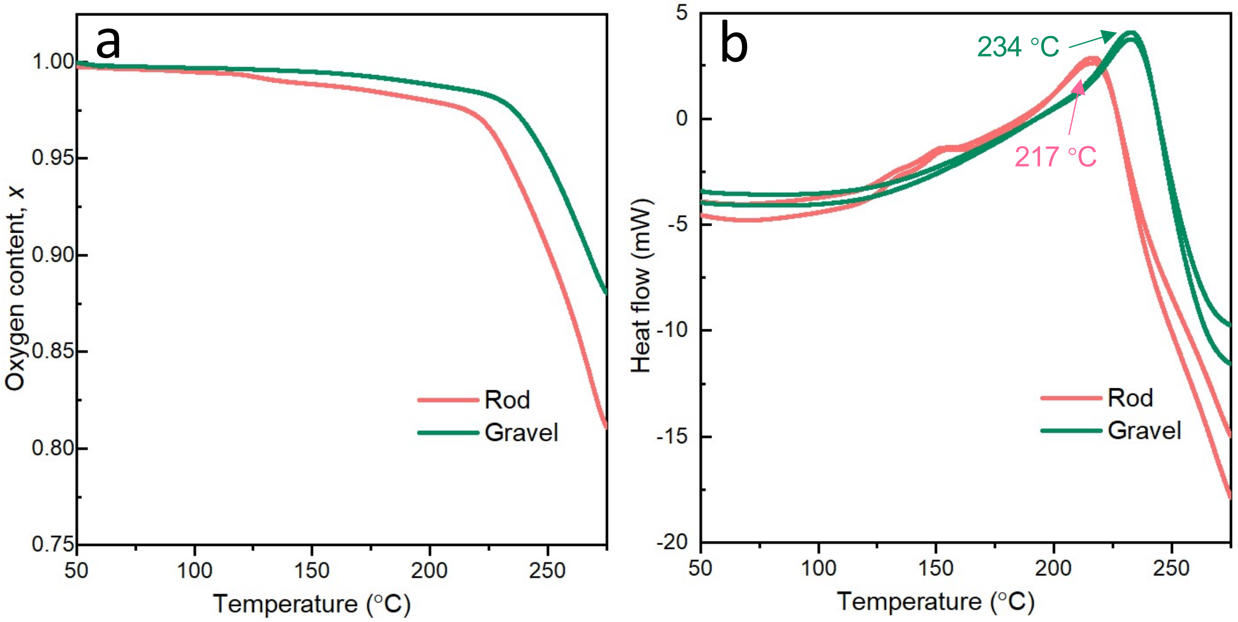

Supplementary Figure 12 Thermal analysis of delithiated NMCs. (a) Estimated oxygen content *x* calculated from TGA datasets by assuming a nominal composition of $Li_{0.3}TMO_{2x}$. **(b)** Extra DSC measurements on chemically delithiated NMC samples, in consistent with the TGA results.

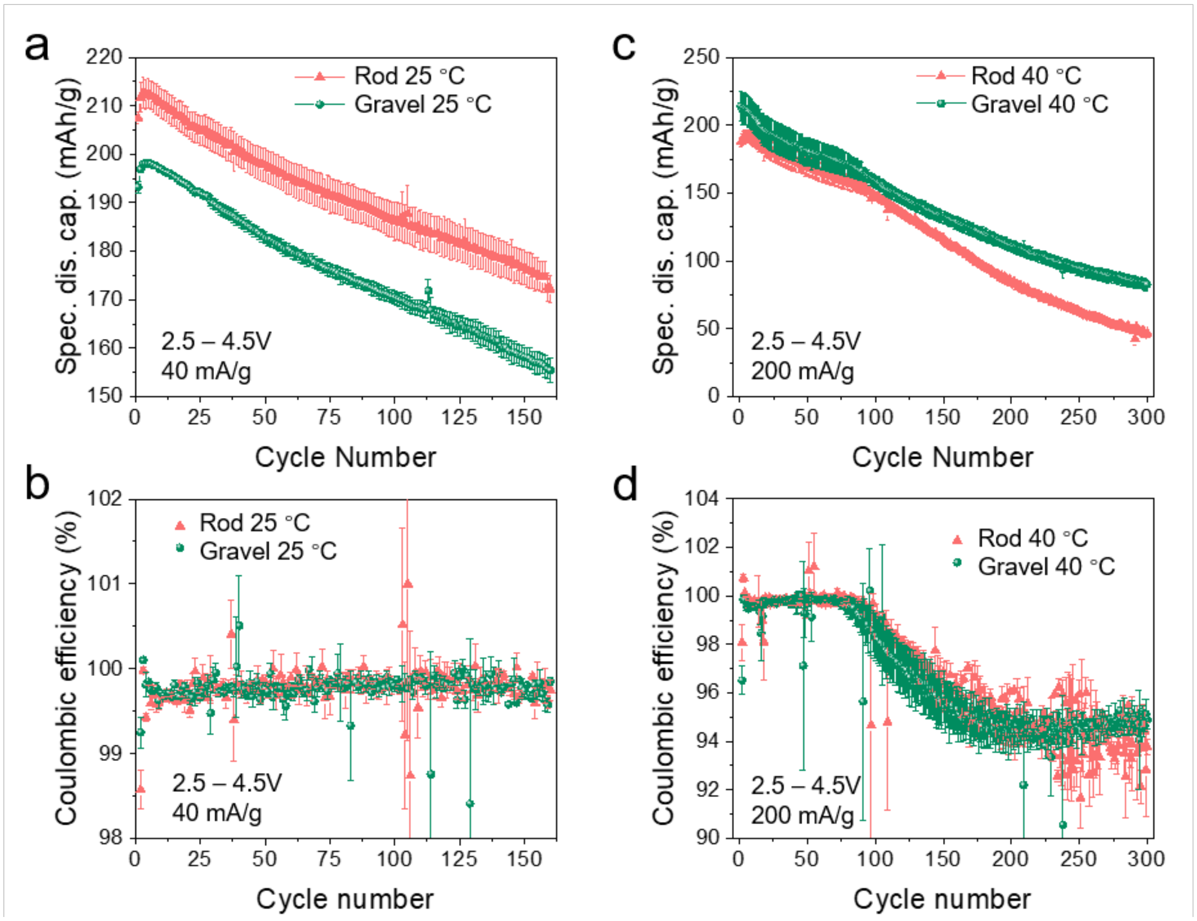

Supplementary Figure 13 (**a**) Specific discharge capacity and (**b**) Coulombic efficiency of gravel- and rod-NMC cells at 40 mA/g (0.2C), 25°C. (**c**) Specific discharge capacity and (**d**) Coulombic efficiency of cells at 200 mA/g (1C), 40°C. Measurements performed on coin cells between 2.5-4.5 V, using lithium metal as negative electrode. The error bars represent the standard deviation from three independent measurements.

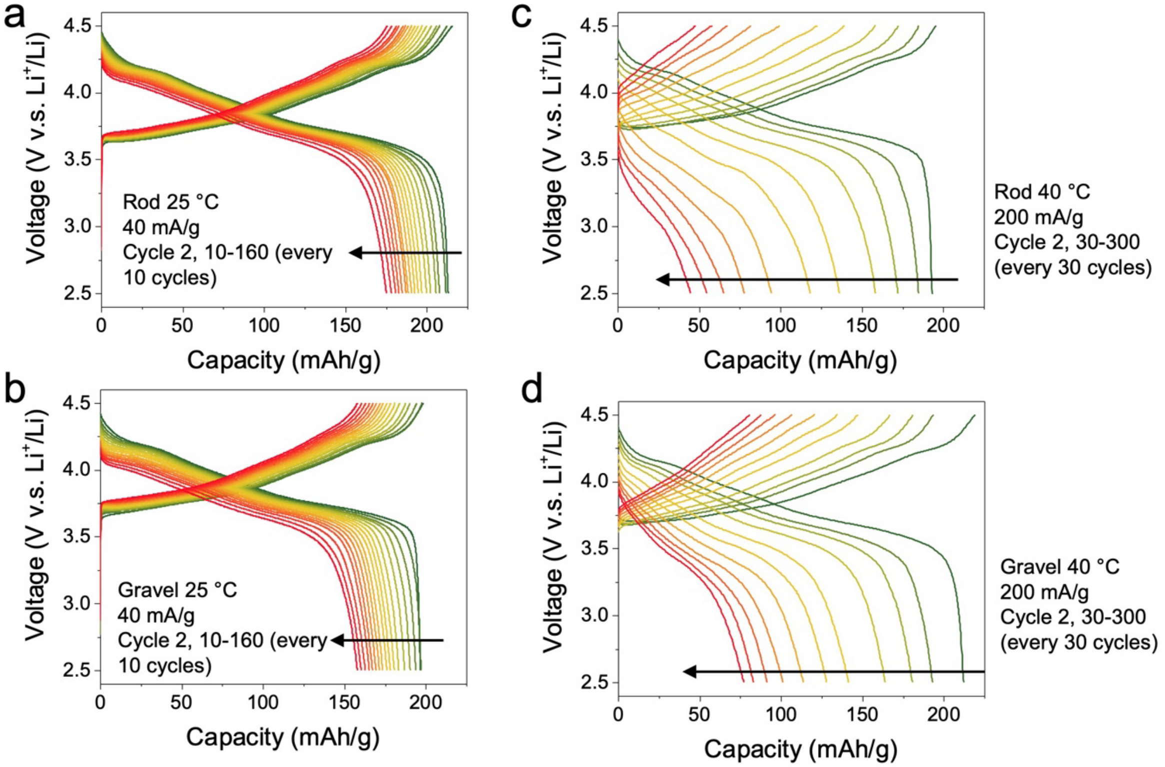

Supplementary Figure 14 Voltage profile during cycling coin cells under different conditions: (a) rod- NMC cells at 40 mA/g (0.2C), 25°C, (**b**) gravel- NMC cells at 40 mA/g (0.2C), 25°C, (**c**) rod- NMC cells at 200 mA/g (1C), 40°C, (**d**) gravel- NMC cells at 200 mA/g (1C), 40°C. Measurements performed on coin cells between 2.5-4.5 V, using lithium metal as negative electrode.

Supplementary Table:

Supplementary Table 1 Crystallographic information of pristine gravel- and rod-NMCs after simultaneous refinement using SXRD and ND patterns.

Gravel $R\overline{3}m$, a = b = 2.87375(2); c = 14.2153(1), wRp: 5.02%, Rp: 4.18%

atom	site	x	y	z	fraction	U_{iso}
Li	3a	0	0	0	0.970(3)	0.0126(6)
Ni	3a	0	0	0	0.009(1)	0.0126(6)
Ni	3b	0	0	0.5	0.791(1)	0.0021(1)
Li	3b	0	0	0.5	0.009(1)	0.0021(1)
Co	3b	0	0	0.5	0.106(7)	0.0021(1)
Mn	3b	0	0	0.5	0.088(8)	0.0021(1)
O	6c	0	0	0.24161(5)	1.009(2)	0.0080(2)

Rod $R\overline{3}m$, a = b = 2.87328(3); c = 14.1996(2), wRp: 4.47%, Rp: 3.25%

atom	site	x	y	z	fraction	U_{iso}
Li	3a	0	0	0	0.977(2)	0.0087(6)
Ni	3a	0	0	0	0.011(1)	0.0087(6)
Ni	3b	0	0	0.5	0.789(1)	0.0032(1)
Li	3b	0	0	0.5	0.011(1)	0.0032(1)
Co	3b	0	0	0.5	0.119(8)	0.0032(1)
Mn	3b	0	0	0.5	0.076(9)	0.0032(1)
O	6c	0	0	0.24139(5)	1.011(2)	0.0080(2)

*Note site fraction is not restricted to stoichiometry.

Supplementary Table 2 Li content and TM ratios from ICP-MS for pristine and delithiated NMCs

Sample name	Cation ratios
Rod-NMC (Pristine)	$Li_{0.989}Ni_{0.821}Mn_{0.060}Co_{0.120}$
Gravel-NMC (Pristine)	$Li_{0.993}Ni_{0.796}Mn_{0.102}Co_{0.102}$
Rod-NMC (120% NO_2BF_4)	$Li_{0.291}Ni_{0.818}Mn_{0.062}Co_{0.120}$
Gravel-NMC (120% NO_2BF_4)	$Li_{0.298}Ni_{0.796}Mn_{0.102}Co_{0.102}$

Supplementary Table 3 Estimated Ni valence states corresponding to the mean white line energy values of XANES-3DTXM histogram at different temperatures. The range of Ni oxidation state in the samples is defined by assuming the highest Ni oxidation state in local domains after charging at 25ºC is Ni^{4+} and the lowest Ni oxidation after heating to 250ºC is $Ni^{2.667+}$ (complete transformation to spinel TM_3O_4)

Temperature (˚C)	Gravel		Rod	
	Mean value (eV)	Oxidation state	Mean value (eV)	Oxidation state
25	8350.4	3.927	8350.5	3.963
150	8349.4	3.537	8348.4	3.090
200	8348.1	2.941	8348	2.889
250	8347.8	2.782	8347.8	2.782

Supplementary Table 4 Crystallographic information of delithiated gravel- and rod-NMCs at 25°C after simultaneous refinement using SXRD and ND patterns.

Gravel $R\bar{3}m$, $a = b$ = 2.8315(6); c = 14.438(3), wRp: 3.82%, Rp: 3.33%

atom	site	x	y	z	fraction	U_{iso}
Li	3a	0	0	0	0.269(3)	0.0103(6)
Ni	3a	0	0	0	0.012(1)	0.0103(6)
Ni	3b	0	0	0.5	0.764(1)	0.0047(3)
Li	3b	0	0	0.5	0.012(1)	0.0047(3)
Co	3b	0	0	0.5	0.105(8)	0.0047(3)
Mn	3b	0	0	0.5	0.094(9)	0.0047(3)
O	6c	0	0	0.23491(5)	0.997(5)	0.0040(2)

Rod $R\bar{3}m$, $a = b$ = 2.8380(7); c = 14.236(3), wRp: 5.88%, Rp: 4.78%

atom	site	x	y	z	fraction	U_{iso}
Li	3a	0	0	0	0.283(2)	0.0118(6)
Ni	3a	0	0	0	0.010(2)	0.0118(6)
Ni	3b	0	0	0.5	0.748(5)	0.0162(8)
Li	3b	0	0	0.5	0.010(2)	0.0162(8)
Co	3b	0	0	0.5	0.119(8)	0.0162(8)
Mn	3b	0	0	0.5	0.076(7)	0.0162(8)
O	6c	0	0	0.23480(3)	0.994(4)	0.0188(6)

*Note site fraction is not restricted to stoichiometry.

Supplementary Table 5 Crystallographic information of delithiated gravel-NMC at 150˚C after refinement on *in situ* ND pattern. wRp: 5.82%, Rp: 4.48%

Layered phase fraction 95.8(2)%, $R\overline{3}m$, a = b = 2.8379(2); c = 14.305(7)

atom	site	x	y	z	fraction	U_{iso}
Li	3a	0	0	0	0.269	0.0163(6)
Ni	3a	0	0	0	0.051(6)	0.0163(6)
Ni	3b	0	0	0.5	0.725(3)	0.0098(2)
Li	3b	0	0	0.5	0.051(6)	0.0098(2)
Co	3b	0	0	0.5	0.105	0.0098(2)
Mn	3b	0	0	0.5	0.094	0.0098(2)
O	6c	0	0	0.2343(5)	0.994(3)	0.0033(9)

Spinel phase fraction 4.2(2)%, $Fd\overline{3}m$, a = b = c = 8.0819(6)

atom	site	x	y	z	fraction	U_{iso}
Li	8a	0.125	0.125	0.125	0.538	0.020*
Ni	16d	0.5	0.5	0.5	0.776	0.0112(1)
Co	16d	0.5	0.5	0.5	0.105	0.0112(1)
Mn	16d	0.5	0.5	0.5	0.094	0.0112(1)
O	32e	0.2518(8)	0.2518(8)	0.2518(8)	1.003(8)	0.0088(2)

*Note Li thermal factors U_{iso} in spinel phase were fixed to an upper limit at elevated temperatures, otherwise, the refinement cannot converge. The cation occupancies were fixed to the values obtained from simultaneous refinement on SXRD and ND patterns at 25˚C.

Supplementary Table 6 Crystallographic information of delithiated rod-NMC at 150˚C after refinement on *in situ* ND pattern. wRp: 5.36%, Rp: 4.43%

Layered phase fraction 12.5(1)%, $R\overline{3}m$, *a* = *b* = 2.8880(10); *c* = 14.190(3)

atom	site	*x*	*y*	*z*	fraction	U_{iso}
Li	3a	0	0	0	0.283	0.0115(6)
Ni	3a	0	0	0	0.0741(1)	0.0115(6)
Ni	3b	0	0	0.5	0.6839(5)	0.0101(5)
Li	3b	0	0	0.5	0.0741(1)	0.0101(5)
Co	3b	0	0	0.5	0.119	0.0101(5)
Mn	3b	0	0	0.5	0.076	0.0101(5)
O	6c	0	0	0.2401(1)	0.980(5)	0.0204(4)

Spinel phase fraction 87.5(4)%, $Fd\overline{3}m$, *a* = *b* = *c* = 8.1062(7)

atom	site	*x*	*y*	*z*	fraction	U_{iso}
Li	8a	0.125	0.125	0.125	0.566	0.020*
Ni	16d	0.5	0.5	0.5	0.748	0.0112(1)
Co	16d	0.5	0.5	0.5	0.119	0.0112(1)
Mn	16d	0.5	0.5	0.5	0.076	0.0112(1)
O	32e	0.2560(9)	0.2560(9)	0.2560(9)	0.991(5)	0.0196(2)

*Note Li thermal factors U_{iso}in spinel phase were fixed to an upper limit at elevated temperatures, otherwise, the refinement cannot converge. The cation occupancies were fixed to the values obtained from simultaneous refinement on SXRD and ND patterns at 25˚C.

Supplementary Table 7 Crystallographic information of delithiated gravel- and rod-NMCs at 250˚C after refinement on *in situ* ND patterns. The final refined spinel phase has a nominal composition of $Li_{0.269}Ni_{0.776}Co_{0.105}Mn_{0.094}O_{1.906}$ for gravel-NMC after 250˚C, while $Li_{0.283}Ni_{0.748}Co_{0.119}Mn_{0.076}O_{1.814}$ for rod-NMC.

Gravel $Fd\overline{3}m$, *a* = *b* =8.2035(4), wRp: 6.15%, Rp: 4.96%

atom	site	*x*	*y*	*z*	fraction	U_{iso}
Li	8a	0.125	0.125	0.125	0.538	0.020*
Ni	16d	0.5	0.5	0.5	0.776	0.0112(1)
Co	16d	0.5	0.5	0.5	0.105	0.0112(1)
Mn	16d	0.5	0.5	0.5	0.094	0.0112(1)
O	32e	0.2532(3)	0.2532(3)	0.2532(3)	0.953(3)	0.0096(7)

Rod $Fd\overline{3}m$, *a* = *b* = 8.2480(4), wRp: 3.98%, Rp: 2.68%

atom	site	*x*	*y*	*z*	fraction	U_{iso}
Li	8a	0.125	0.125	0.125	0.566	0.020*
Ni	16d	0.5	0.5	0.5	0.748	0.0129(2)
Co	16d	0.5	0.5	0.5	0.119	0.0129(2)
Mn	16d	0.5	0.5	0.5	0.076	0.0129(2)
O	32e	0.2473(5)	0.2473(5)	0.2473(5)	0.907(5)	0.0118(1)

*The cation occupancies were fixed to the values obtained from simultaneous refinement on SXRD and ND patterns at 25˚C.

Reference List:

1. Cao, C. *et al.* Emerging X-ray imaging technologies for energy materials. *Mater. Today* **34**, 132–147 (2020).
2. Ge, M. *et al.* One-minute nano-tomography using hard X-ray full-field transmission microscope. *Appl. Phys. Lett.* **113**, 083109 (2018).
3. Otsu, N. A Threshold Selection Method from Gray-Level Histograms. *IEEE Trans. Syst. Man. Cybern.* **9**, 62–66 (1979).
4. Tian, C. *et al.* Charge Heterogeneity and Surface Chemistry in Polycrystalline Cathode Materials. *Joule* **2**, 464–477 (2018).
5. Yin, S.-C., Rho, Y.-H., Swainson, I. & Nazar, L. F. X-ray/Neutron Diffraction and Electrochemical Studies of Lithium De/Re-Intercalation in $Li_{1-x}Co_{1/3}Ni_{1/3}Mn_{1/3}O_2$ (x = 0 → 1). *Chem. Mater.* **18**, 1901–1910 (2006).
